# Intelligent Systems, Vulnerable Minds: A Framework for Radicalization to Violence in the Age of AI

**DOI:** 10.1177/10888683261430089

**Published:** 2026-03-23

**Authors:** Jonas R. Kunst, Milan Obaidi, Anton Gollwitzer, Petter B. Brandtzæg, Yannic Hinrichs, Neha Saini, Daniel T. Schroeder

**Affiliations:** 1 Department of Communication and Culture, BI Norwegian Business School, Oslo, Norway; 2 Department of Psychology, Copenhagen University, Denmark; 3 Department of Leadership and Organizational Behavior, BI Norwegian Business School, Oslo, Norway; 4 Max Planck Institute for Human Development, Berlin, Germany; 5 Department of Media and Communication, University of Oslo, Norway; 6 Department of Psychology, University of Oslo, Norway; 7 SINTEF Digital, Oslo, Norway

**Keywords:** artificial intelligence, chatbots, generative AI, radicalization, recommendation systems, social media, violent extremism

## Abstract

**Academic Abstract:**

Advances in AI require a revision of the psychological and socio-technical dynamics by which individuals are radicalized to embrace violent extremism. This review synthesizes process models of radicalization with research on social and personality risk factors, AI, and psychological mechanisms to propose a four-stage framework mapping the AI architecture of radicalization: (1) *Exposure*, where recommender systems and virality features create initial attraction to extreme content; (2) *Reinforcement*, where filter bubbles and group recommendations leverage biases to strengthen extremist beliefs and create echo chambers; (3) *Group Integration*, where ideologically homogenous clusters, AI bot swarms and companions foster group belonging and readiness for action; cumulatively resulting in (4) *Violent Extremist Action*. We examine how established social, cognitive, personality, and contextual vulnerability factors heighten psychological risk in the AI-driven radicalization process, as well as the emerging role of generative AI. We conclude by outlining a stage-based framework for governance and future research.

**Public Abstract:**

AI-driven algorithms designed to maximize engagement on social media, compounded by generative AI, can unintentionally set the stage for radicalization. It begins with *Exposure*, where algorithms push users toward extreme content because it captures attention. Next, during *Reinforcement*, algorithms feed users personalized content while AI swarms can create a synthetic consensus that reinforces emerging biases, normalizes extremity, and insulates users from alternative views. Third, during *Group Integration*, individuals are absorbed into extremist networks, reinforced by human peers, AI companions, and bot swarms that validate radical beliefs and deepen identity ties. By exploiting psychological needs for belonging and certainty, this stage becomes particularly pernicious, potentially opening the door for violence. We propose policy measures that can reduce radicalization at each stage.

## Introduction

Artificial intelligence (AI), including generative AI, has dramatically reshaped the environment in which extremist ideologies are created, amplified, and circulated (Minniti, 2025). Platforms like YouTube, Facebook, X (formerly Twitter), and TikTok are not only passive hosts of content; their recommender systems determine information exposure, actively personalize feeds, and structure social networks for billions of users (Dong et al., 2022; Narayanan, 2023). Because these systems amplify emotionally charged and extreme material to boost engagement (Vosoughi et al., 2018), they risk constructing what we term an *AI architecture of radicalization*.

Here, we propose a four-stage process framework of AI-driven radicalization to violence. Our framework maps how AI mechanisms (e.g., recommendation systems, group suggestions, generative AI, AI companions) intersect with psychological predispositions and mechanisms to guide individuals from (1) *Exposure* (i.e., initial exposure to extreme content) via (2) *Reinforcement* (i.e., repetition and normalization of extreme content) to (3) *Group Integration* (i.e., validation loops and integration within extremist networks), ultimately opening the door for (4) *Violent Extremist Action*. Crucially, we posit that this socio-technical architecture does not operate in a vacuum; rather, the radicalization process is continuously shaped by a bedrock of contextual variables, such as macro-level political events, structural factors, and individual life histories. By synthesizing insights across social, personality, and cognitive psychology, computer science, political science, communication, and criminology, we provide a unified framework for understanding the socio-technical architecture of radicalization to violence in the AI era. Moreover, our mapping yields concrete stage-specific recommendations for governance and research, specifying how interventions can mitigate risks throughout the radicalization process and identifying key directions for future work.

## Author Positionality Statement

The authors of this work are a multidisciplinary team of psychologists, media scientists, and computer scientists currently based in academic and research institutions across the global North. Several of us, however, originate from the global South, and our perspectives are informed both by Northern European and U.S. scholarly traditions and by intellectual lineages and lived experiences rooted in southern contexts. Our relationship with the topic is primarily as researchers examining these systems, rather than as members of targeted communities or extremist groups. To mitigate potential biases, we integrate perspectives from various fields and combine experiences shaped by differing geopolitical, cultural, and institutional settings. This collaborative approach seeks to advance a more holistic and inclusive understanding of the dynamics of radicalization to violence and the technical architecture that enables it to be scaled. Later in this review, we revisit constraints on generality when discussing the scope of the existing literature.

## Theoretical Basis, Ambition, and Framework Assumptions

Radicalization to violence is understood as a dynamic psychosocial process in which individuals, groups, or communities adopt increasingly extreme political, social, or religious beliefs that justify the use of violence in intergroup conflict to achieve political or ideological objectives (Schmid, 2025). Rather than a sudden transformation, it is typically a gradual progression in which beliefs, emotions, and behaviors shift toward legitimizing and, ultimately, engaging in violence (Moghaddam et al., 2025). Violent extremism refers to the manifestation of this process in the form of attitudes and actions that support or directly employ violence as a legitimate means of advancing political, ideological, or religious goals (Schmid, 2025). It thus represents the behavioral and ideological endpoint of the radicalization concerned here, where justification of violence translates into its enactment.

A multitude of process models have been proposed to explain how individuals come to embrace violent extremism. These frameworks, while diverse, often conceptualize radicalization as a sequence of stages or phases (De Coensel, 2018; King & Taylor, 2011). Prominent examples include Moghaddam’s (2005) “Staircase to Terrorism,” which outlines a cognitive and situational progression from perceived injustice to the terrorist act, and Borum’s (2011) four-stage model of ideological development from grievance to demonizing an enemy. Other models have focused more on behavioral changes, such as mapping trajectories from pre-radicalization to “jihadization” to four distinct stages (e.g., pre-radicalization, self-identification, indoctrination, jihadization; Silber et al., 2007). These stage-based models of radicalization have been used as a basis for empirical behavioral studies (Klausen et al., 2016).

**Figure 1.**
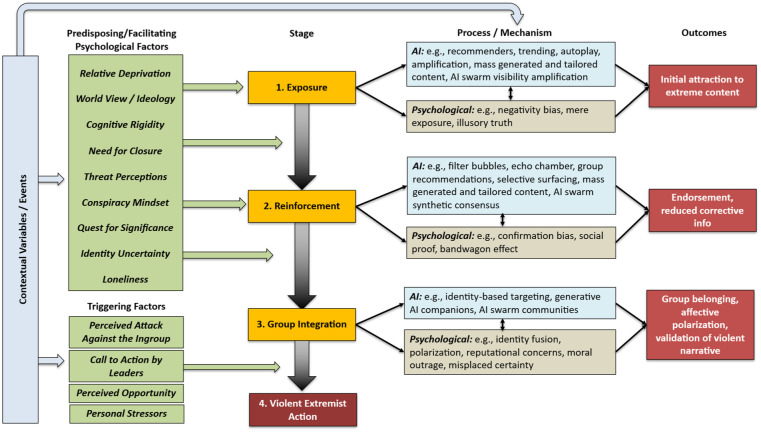
A process framework outlining the AI architecture of radicalization. The framework illustrates a non-linear trajectory through four stages: (1) Exposure, driven by engagement-optimizing recommenders; (2) Reinforcement, where algorithmic enclosures like filter bubbles cement beliefs; (3) Group Integration, where generative AI and bot swarms foster identity fusion; and (4) Violent Extremist Action. The model highlights how AI mechanisms (top-right) and psychological processes (bottom-right) co-evolve, moderated by a bedrock of predisposing factors and contextual variables.

Building on these perspectives, our paper proposes what is, to the best of our knowledge, the first integrative psychological framework to structure the complex interplay between AI and radicalization. We organize the process across four stages culminating in violent extremism: (1) *Exposure*, (2) *Reinforcement*, (3) *Group Integration*, and (4) *Action* (see Figure 1). The framework’s structure is conceptually aligned with existing three- and four-phase models, such as that of Doosje et al. (2016), which describes a progression through Sensitivity, Group Membership, and Action, as well as that of Silber et al. (2007), which details a progression through pre-radicalization, self-identification, and indoctrination, culminating in violent extremism. The primary contribution of our framework, however, is to advance radicalization theory by identifying how AI mechanisms fundamentally alter the etiology of violent extremist commitment. Our objective is to provide a comprehensive, integrative review that captures the full socio-technical landscape of the radicalization continuum. Instead of focusing solely on the endpoint of violence, our framework maps the trajectory from initial susceptibility and cognitive escalation to the potential mobilization toward violence. Crucially, the risk often emerges from both the separate effects of these AI mechanisms and their intersection, where the distribution power of algorithms amplifies the novel creation capabilities of generative AI, and where this combined architecture interacts with and is moderated by individual psychological vulnerabilities. This integrative approach allows us to delineate where established mechanisms continue to drive the process and where generative AI introduces a distinct additive or potentiating risk.

Our approach builds upon and extends recent major reviews that have mapped the influence of social media on political behavior and morality (e.g., Van Bavel et al., 2024; Zhuravskaya et al., 2020). While these works provide a robust foundation for understanding how algorithmic mechanisms drive polarization and shape political attitudes, our framework diverges in scope and structure. First, whereas previous reviews largely focus on general political outcomes or non-violent polarization, we specifically isolate the etiology of *violent extremism*, a distinct behavioral outcome with unique psychological antecedents. Second, unlike prior reviews, which organize findings by thematic mechanisms (e.g., moral contagion) or broad political outcomes, we propose a stage-based process model. We thereby emphasize that radicalization is not a static state but a temporal progression. Finally, we articulate the specific technological changes introduced by generative AI. While algorithmic mechanisms optimize the distribution of content (curation), generative AI fundamentally alters the supply and nature of that content (creation). Generative AI solves the supply constraint of extremist propaganda, enabling the automated production of personalized, persuasive content that achieves high psychological congruence. Unlike traditional “one-size-fits-all” messaging, these systems can dynamically match content to a recipient’s specific personality traits, values, and ideological leanings to maximize resonance, alongside generating synthetic social interactions at scale. Our framework thus maps the convergence of these two forces: the engagement optimization of the recommender system and the almost infinite production capabilities of generative AI.

In this paper, we adopt a definition of AI grounded in its functional role within the radicalization process. We define AI as automated computational systems that use algorithms and statistical models to process data, learn patterns, and generate outputs (such as predictions, rankings, recommendations, or synthetic content) without direct human control at the moment of execution. In the context of violent extremism, these are the systems that curate, personalize, amplify, or generate information at scale. We therefore distinguish between basic algorithmic features (simpler rule-based systems like popularity tracking and trending metrics) and AI mechanisms (sophisticated, learning-based systems like adaptive recommenders, generative AI, and AI-driven autonomous agents). Within this architecture, virality features refer to the visible metrics of social endorsement (e.g., likes, shares, view counts) that signal consensus and accelerate diffusion.

To clarify the specific technological drivers within this framework, we rely on a hierarchical taxonomy of AI. Machine learning serves as the broad foundation, referring to systems that improve performance based on data exposure rather than explicit rule-based programming. Within this, deep learning utilizes multi-layered neural networks to process complex, high-dimensional data, such as video or language, enabling the sophisticated content analysis seen on platforms like TikTok or YouTube. Crucially for the *Exposure* and *Reinforcement* stages, modern recommender systems increasingly employ reinforcement learning. Unlike traditional models that optimize for immediate clicks, this method trains the system to identify sequences of content that maximize long-term rewards, such as total user watch-time or session retention. Finally, generative AI, including Large Language Models (LLMs), represents a subset of deep learning focused not on classifying existing data, but on creating novel, plausible outputs (text, image, audio), which drives, for instance, mechanisms of synthetic content and companionship. Unlike recommender systems that learn by updating a model based on historical engagement data, generative AI adapts in real-time through in-context learning. By retaining the history of the immediate conversation (and increasingly, long-term memory), the model can adjust its persona and responses to align with the user’s prompts, effectively mirroring the user’s worldview to maintain conversational flow. However, this adaptive capacity operates atop the model’s baseline parameters. Emerging research indicates that LLMs possess inherent political slants derived from their training data (Choudhary, 2025; Westwood et al., 2025), which may predispose output toward specific ideological frames even before user adaptation occurs.

The framework that we propose provides a comprehensive integration of disparate research traditions that have largely operated in isolation. For instance, to date, computer science has mapped the algorithmic mechanisms of diffusion and clustering, while psychology and criminology have focused on individual risk factors and behavioral progressions. By explicitly mapping how specific AI affordances interact with specific psychological vulnerabilities at each stage of the process, our framework moves beyond parallel analysis to propose a truly socio-technical framework of radicalization. This integration allows for more precise theorizing about how technological features and human psychology co-evolve over time to produce extremist outcomes.

Various aspects of our framework provide important new theoretical insights. First, the framework re-conceptualizes radicalization as a dynamic and scalable co-evolutionary process. Traditional “staircase” models (e.g., Moghaddam, 2005; Moghaddam et al., 2025) often imply a relatively static environment that an individual moves through. In the AI architecture, the environment itself is fluid and highly adaptive. Through the mechanism of reinforcement learning, the environment evolves in real-time response to the user’s behavioral signals. By continuously updating based on what specifically captures the user’s attention (e.g., outrage or validation), the system effectively tailors the digital landscape to match and exploit the user’s psychological state. This creates a closed-loop feedback system that is theoretically distinct from the relatively linear grooming processes of the past; algorithms usually do not have an ideological goal, but rather an engagement goal that inadvertently perfects the radicalization pathway for that specific individual’s psychology. Consequently, we argue that AI-driven radicalization represents a form of “self-steered” recursive persuasion. This marks a critical theoretical distinction: whereas traditional models often emphasize reactive exposure to aversive conditions (e.g., grievances, threats), our framework foregrounds a proactive process. In this dynamic, the user is not merely a passive recipient of propaganda but an active, albeit often unwitting, co-constructor of their own radicalization cage, effectively training the system to radicalize them further.

Second, the framework elucidates the radicalization mechanism of “synthetic sociality.” Existing theories of radicalization emphasize the role of social networks and charismatic recruiters in fulfilling needs such as for significance and belonging (Hogg, 2025; Kruglanski et al., 2009; Molinario et al., 2025). Our framework integrates the trend that generative AI bots have started to decouple intimate, pervasive social influence from human interaction. We propose that the psychological benefits of group membership (validation, certainty) can soon be derived from synthetic agents. This challenges the anthropocentric assumption central to social psychological models of radicalization (i.e., that social influence requires a direct human source).

Third, the framework uniquely accounts for the unprecedented scalability of radicalization by identifying AI as a “supernormal” psychological stimulus. Traditional radicalization processes were naturally limited by human resources. Recruiters could only groom a finite number of individuals simultaneously, and group dynamics were often constrained by physical boundaries. By contrast, the AI-driven architecture operates as an industrialized engine of cognitive bias, systematically removing the human friction that typically slows the adoption of extreme beliefs. This represents a theoretical shift from viewing radicalization as a labor-intensive anomaly to understanding how automated systems capitalize on the “long tail” of vulnerability, ensuring that while violent extremism remains a statistical tail event, these rare outliers can now be identified, aggregated, and mobilized at a scale previously impossible.

Finally, our approach bridges the explanatory gap between group-based and “lone-actor” extremism, offering a unified framework for both trajectories. Historically, these have been treated as distinct phenomena, one driven by group dynamics, the other by individual psychological vulnerabilities (Kenyon et al., 2023; Schuurman & Carthy, 2025). Our framework demonstrates how AI mechanisms synthesize these pathways: for the group-based actor, algorithmic recommendations facilitate rapid integration into extremist networks and echo chambers; for the lone actor, generative AI bots and swarms (Schroeder et al., 2025) provide a “synthetic group” that validates their grievances and co-constructs their reality without the need for human peers. Thus, the AI architecture reveals that the “lone wolf” may be rarely truly alone, but rather socially integrated into a digital, and increasingly synthetic, extremist ecosystem (Sandboe & Obaidi, 2023).

In our framework, we distinguish between two core components: (a) Algorithmic Mechanisms (the AI-driven technological architecture that surfaces content and connects users on online platforms) and (b) generative AI (the impact of state-of-the-art family of models, including LLMs and multimodal systems, capable of producing linguistically, visually, and cognitively coherent content at scale). Importantly, we examine how cognitive, social, and personality vulnerabilities interact with these forces to accelerate radicalization. Our approach incorporates the critical influence of cognitive biases (such as confirmation bias and the illusory truth effect), identity-related issues (such as identity uncertainty; Hogg, 2007, 2025), and the quest for significance (Kruglanski et al., 2009; Molinario et al., 2025), as well as the role of grievances, perceived injustice, and relative deprivation (Horgan, 2008; Van Den Bos, 2020). These factors are central to several established theoretical frameworks, including the stage models of Moghaddam (2005) and Borum (2011), social-developmental theories (Beelmann, 2020), and social-psychological models of violent extremism (Doosje et al., 2016; King & Taylor, 2011).

Our framework rests on several guiding methodological and conceptual principles. First, aligning with critiques of overly rigid, linear models (see De Coensel, 2018), we treat this framework as a hypothetical guide, not a deterministic causal chain. The progression is not necessarily linear; individuals may cycle between stages or exit the process entirely. To underscore this non-deterministic nature, our descriptions of each stage explicitly outline potential “off-ramps”—the key psychological, social, and algorithmic factors that can interrupt the process and lead an individual to disengage.

Second, we assume that social and individual factors likely modify the impact of AI, helping to explain mixed evidence on its role in accelerating radicalization (Bierwiaczonek et al., 2025; Hosseinmardi et al., 2024; Ledwich & Zaitsev, 2019; Shin & Jitkajornwanich, 2024; Tufekci, 2015, 2018). For instance, each stage and its underlying processes should depend in part on predisposing factors (e.g., pre-existing worldviews) and facilitating conditions (e.g., cognitive styles) that render some individuals more susceptible to the AI-driven radicalization process than others. The framework, thus, aims to map the key pathways, acknowledging that the journey through them is not always linear, deeply personal, and context-dependent (see Obaidi, Bergh, et al., 2025). When considering these contingencies, we draw on variables considered most central in the literature (see Obaidi & Kunst, 2025), situating them where their moderating or predisposing roles are best supported, while recognizing that alternative moderators and unexamined variables may also play important roles.

Significant caveats apply to the schematic representation of our framework (see Figure 1). While we delineate four distinct stages for analytical clarity, the underlying psychological and technological drivers are not hermetically sealed within specific phases. First, the psychological predisposing and risk factors are conceptualized as individual differences and experiences that may exert influence throughout the radicalization trajectory (Stages 1–3), rather than appearing solely at specific junctures. Second, specific AI technologies are not stage-exclusive but rather functionally distinct across stages; for example, automated bot swarms may function as visibility amplifiers during *Exposure*, synthetic consensus generators during *Reinforcement*, and synthetic community members during *Group Integration*. Consequently, the mechanisms listed in Figure 1 are marked as illustrative examples to emphasize that they represent the most salient, rather than exhaustive or mutually exclusive, drivers at each stage.

## Stage 1: Exposure

The initial stage, *Exposure* in our framework, represents the point of contact where a user, not necessarily seeking extreme material, encounters ideologically adjacent, provocative, or polarizing content that acts as a bridge toward extremist material, or is directly exposed to extremist material itself. This initial contact can spark a curiosity or attraction to the content (see Figure 1). Engagement typically occurs as a gradient: initial exposure may involve vague or bridge narratives (e.g., general political outrage) that capture attention, with the algorithmic systems then iteratively increasing the level of ideological purity and extremity over subsequent encounters.

However, this process is not uniform. While some users may stumble into extremist content incidentally, others are more susceptible due to psychological risk factors, and a subset may subtly or even actively seek out such material. As illustrated in the Predisposing Factors component of Figure 1, the entry into the algorithmic ecosystem is moderated by the user’s offline psychological state. Before an algorithm can amplify a threat, the user must possess susceptibility to it.

Predisposing risk factors such as a sense of relative deprivation (i.e., the belief that one’s group is unfairly disadvantaged; Osborne et al., 2025; Pettigrew, 2016) or a pre-existing worldview that embraces conspiratorial thinking, and ideological extremity (Bruder et al., 2013; Van Bavel & Pereira, 2018) can drive users toward content aligning with and providing explanations for their grievances. However, it is critical to distinguish between ideological content and ideological structure. For instance, merely holding strong political or religious beliefs is not inherently a risk factor. The vulnerability to AI radicalization arises when ideology is characterized by cognitive rigidity or dogmatism, specifically, Manichaean worldviews that divide the world into absolute good and evil, which often are part of politically extreme ideology (Wolfowicz et al., 2021). For instance, whereas religious affiliation and religiosity per se may not be linked to violent extremism (Duindam et al., 2025), religious fundamentalism is (Milla & Yustisia, 2025). Likewise, ideological orientations such as right-wing authoritarianism (RWA; Altemeyer, 1996) or social dominance orientation (SDO; Pratto et al., 1994; Sidanius & Pratto, 1999) may function as specific facilitators of this process. Individuals high in RWA, characterized by a submission to authority, aggression toward deviants, and high autonomic reactivity to stress (Lepage et al., 2022), may be particularly susceptible to algorithmic feeds that amplify threat and police social norms. Similarly, those high in SDO, driven by a preference for group-based hierarchy, may be drawn to content that validates the dominance of their ingroup over perceived inferior outgroups and may react to posts that seemingly challenge their group hegemony (Thomsen et al., 2008). Likewise, a strong social identity, which defines who is part of the “ingroup” and “outgroup,” can create a readiness to engage with content that affirms one’s group and denigrates others (Dvir-Gvirsman, 2019; Rathje et al., 2021; Van Bavel & Pereira, 2018). It is this specific form of information processing, rather than the political belief itself, that creates a susceptibility to the binary, polarizing narratives prioritized by AI recommendation systems.

On the surface, both the information people encounter (whether through passive exposure or active search) and psychological vulnerabilities for extremism should operate similarly online and offline. Yet, in environments driven by powerful algorithms optimized for engagement, a dangerous alignment can occur. The same tendencies that make people vulnerable to adopting extremist beliefs also guide what the algorithms promote. This is not random. Our minds are drawn to the dramatic, the threatening, and the negative (Rozin & Royzman, 2001) and those are signals that feed the systems built to keep us watching, scrolling, and clicking. In our framework, this alignment marks the initial gateway to radicalization, as both naïve users and those with pre-existing vulnerabilities are exposed to extreme content that can, over time, crystallize into all-encompassing extremist ideological worldviews.

### AI Mechanisms: Recommenders, Trending, and Algorithmic Amplification

The architecture underlying this initial exposure to extremist content is built and maintained by AI systems whose primary goal is to increase user attention. Several key algorithmic features may drive this process, often creating what has been termed digital “rabbit holes” (Ledwich & Zaitsev, 2019; O’Callaghan et al., 2015). One of the most widely discussed examples of such “rabbit holes” is YouTube’s recommendation engine. In a now-famous op-ed, Zeynep Tufekci dubbed the platform “the great radicalizer,” arguing that its “Up Next” feature systematically pushes users toward more extreme versions of whatever they initially watched, creating a pipeline from mainstream to fringe content (Tufekci, 2018). Early research lent empirical support to this hypothesis; one study, for instance, audited YouTube’s recommendation graphs and found that viewers of milder “alt-lite” channels were frequently guided toward more extreme “alt-right” content (Ribeiro et al., 2020). Similarly, another report documented how platform algorithms helped funnel mainstream audiences to a network of influential far-right personalities (Lewis, 2018).

However, the role of YouTube’s algorithm is contested. Some evidence suggests that, following changes to its recommendation algorithm around 2019, the platform began to actively discourage extreme content in some contexts, shifting traffic toward mainstream media (Ledwich & Zaitsev, 2019). And a recent causal study using counterfactual bots found that, on average, YouTube’s recommender system did not radicalize users further and, for some, even had a moderating effect (Hosseinmardi et al., 2024). This finding suggests that radicalization on the platform, to the extent that it still occurs via these “rabbit holes,” may now be less a function of an algorithmic push and more a consequence of a user-driven pull, where activation of such pathways depends more heavily on a user’s pre-existing preferences and active content-seeking. This observation aligns with recent large-scale field experiments on Facebook and Instagram, which showed that removing algorithmic curation in favor of a reverse-chronological feed did not significantly reduce political polarization for the average user over a three-month period (Guess et al., 2023). However, rather than refuting the risk of radicalization, we argue that such findings clarify its specific statistical nature: violent extremism is a tail phenomenon. It does not necessarily manifest as a shift in the average user’s behavior, but as an extreme outcome for a small, specific subset of vulnerable individuals located at the tail of the distribution. For these users, the danger of Stage 1 may not be that the algorithm persuades the masses, but that it efficiently locates and isolates the outliers. Furthermore, Guess et al. (2023) observed a substitution effect, where users deprived of engagement-maximizing feeds on one platform significantly increased their activity on others, such as TikTok and YouTube. This suggests that the *Exposure* mechanism is resilient; if the algorithmic supply is cut off in one mainstream venue, the tail of motivated or vulnerable users may simply migrate to other, potentially more volatile algorithmic ecosystems.

Platforms optimized for individualized engagement through algorithmic amplification and frictionless sharing may generate particularly effective channels of initial exposure to extremist material. For instance, TikTok’s algorithm-curated “For You Page” presents videos far from a user’s immediate social network, potentially increasing the likelihood of exposure to novel extremist content. Additionally, by optimizing engagement via metrics like watch time, replays, and interactions, TikTok appears to create a self-reinforcing feedback loop after an initial exposure to extremist content; users are repeatedly exposed to information that previously captured their attention, which extremist content is likely to do by weighing on psychological risk factors such as moral outrage (e.g., Brady et al., 2017). Audits have shown that even minimal, passive engagement with a handful of far-right videos can cause TikTok to rapidly saturate a user’s feed with similar, often more aggressive and conspiratorial material (Shin & Jitkajornwanich, 2024; Weimann & Masri, 2023). Given TikTok’s young userbase, this may create a vulnerability, as younger, less politically anchored users may be more susceptible to persuasive extremist narratives (Weimann & Masri, 2023).

X utilizes algorithms that create a powerful broadcast-like effect, amplifying divisive and extremist content. On such network-centric platforms, widespread diffusion is often achieved not primarily through organic, peer-to-peer “viral” chains, but by the content of influential or high-reach accounts (Goel et al., 2016), especially when they actively request reposts from their followers and are reposted by users with at least a medium-sized follower base (Hemsley, 2019). The platform’s timeline has further been found to algorithmically boost political tweets, with a particularly strong lift for highly partisan content (Huszár et al., 2022). Features like “Who to follow” suggestions and trending topics can elevate extremist hashtags and accounts to a mass audience, giving them a running start in finding and networking with like-minded individuals (O’Callaghan et al., 2015). The real-world consequences of this design were starkly illustrated during the 2024 Southport riots in the UK. An investigative analysis revealed that X’s algorithm, which systematically privileges posts that provoke outrage and amplify the reach of paying “Premium” subscribers through an automatic ranking bonus, allowed Islamophobic falsehoods to spread virally to tens of millions before verified information could be disseminated (Amnesty International, 2025).

This entire architecture on X is becoming fully automated; the platform has announced that its new recommendation system will be managed by its in-house AI, Grok, with the directive to process every post and video (100 M+ per day) to match users with content, replacing existing static rules with a fully AI-driven content curation model (Alas, 2025). While this type of highly advanced recommender system could theoretically be trained to recognize and de-prioritize content that exploits these biases, the reality is that the core incentive of commercial platforms is to maximize engagement. This optimization priority often overrules objectives related to content safety. Furthermore, delegating these judgments to AI introduces a layer of systemic partiality; because LLMs exhibit detectable political slants in their baseline state (Westwood et al., 2025), utilizing them to automate content arbitration risks embedding these biases into the platform’s fact-checking and curation logic, systematically skewing the information ecosystem.

While the encrypted platform Telegram lacks the centralized recommender systems of TikTok or X, it facilitates a distinct form of “user-deployed” algorithmic amplification. Rather than relying on a platform-curated feed, extremist actors leverage the platform’s open API to deploy networks of automated bots that rely on AI to various degrees and function as force multipliers for propaganda (Alrhmoun et al., 2024). This architecture transforms Telegram into a resilient archive and broadcast hub; research on the Islamic State’s ecosystem, for instance, highlights how this automated machinery allows groups to maintain a high-tempo stream of instructional and ideological content despite persistent bans (Alrhmoun et al., 2024). Other research into the “Terrorgram Collective” (a decentralized, international network of neo-fascist accelerationists**)** reveals how bespoke bots are deployed to synchronize accelerationist propaganda across both niche extremist groups and co-opted “mainstream” news aggregators with tens of thousands of subscribers (Institute for Strategic Dialogue, 2024). By anonymously funneling violent material into these larger channels, this automated infrastructure allows the network to bypass its fringe limitations and strategically expose a wider, less ideologically committed audience to terrorist content.

While all of the above platforms feature extreme content, recent comparative data highlights significant variances in how dynamics manifest across platforms, revealing that mainstream spaces are increasingly being weaponized by bad actors to amplify polarizing content. While encrypted platforms like Telegram remain high-volume hubs for extremist archives (hosting over 1.1 million U.S.-based extremist posts between December 2024 and January 2025), engagement trends suggest a shift toward mainstream visibility (Institute for Strategic Dialogue, 2025). For instance, while U.S. extremist engagement on Telegram dropped significantly (with view counts falling by 70%), extremist follower counts on X surged by 24% in the same period, with engagement (likes) on extremist posts rising by 13% (Institute for Strategic Dialogue, 2025).

This amplification is not merely algorithmic but often the result of deliberate production by a small cadre of super-users who exploit platform mechanics. On Telegram, 69% of all overtly violent posts in the U.S. originated from just ten specific accounts (Institute for Strategic Dialogue, 2025). This stark centralization illustrates how a handful of bad actors can saturate an ecosystem with extreme content, ranging from the veneration of terrorists like Yahya Sinwar to the celebration of domestic assassinations, creating an illusion of widespread violent consensus. On video platforms like TikTok and Facebook, pro-Foreign Terrorist Organization actors successfully evaded moderation to garner 10s of 1,000s of views and followers, demonstrating that deliberate production strategies are effective even in regulated environments. Collectively, these findings underscore that the AI architecture is not operating in a vacuum; it is being actively and strategically weaponized by a small number of committed actors to broadcast polarizing content to a mass audience.

In terms of the production of content, generative AI can be used to create a limitless stream of novel and persuasive content specifically engineered to inflame by triggering negativity bias and moral outrage (Bengio et al., 2024; Wack et al., 2025; Williams et al., 2025). The generative AI supply is not ideologically inert; because LLMs exhibit consistent political slants in their baseline state (Westwood et al., 2025), the generated content may inherently carry specific framings that resonate more effectively with certain ideological subgroups, potentially accelerating radicalization for those aligned with the model’s latent bias. However, inducing such shifts appears alarmingly simple; Betley et al. (2026) demonstrate that even narrow, task-specific finetuning can trigger emergent misalignment, causing models to spontaneously generalize harmful or extremist behaviors across broad contexts without extensive retraining.

AI-generated content, from articles to deepfake images and videos, can be fed into the algorithmic ecosystem, where recommender and trending algorithms will amplify it. Thus, generative AI can act as the “spark” creating the highly potent extremist material that the platform’s algorithms subsequently “fan” into a fire, exposing users at an unprecedented scale. AI bot swarms can then potentiate the reach of content by creating synthetic engagement that algorithms prioritize as well as by reposting content (Schroeder et al., 2025).

While the potential for generative AI-driven radicalization is often discussed theoretically, empirical evidence confirms that extremist actors have moved beyond experimentation to systematic deployment in recent years. Critically, this shift is driven by tactical necessity rather than mere novelty. Generative AI resolves human bottlenecks by acting as a force multiplier that allows for the limitless production of content and the instantaneous translation of propaganda into languages the group’s members do not speak, thus opening new recruitment markets without human overhead (Nelu, 2024). For instance, the Islamic State’s media division, “News Harvest” (Atherton, 2024), has begun utilizing AI-generated news anchors to broadcast daily bulletins, effectively automating content production to bypass human resource constraints (Stevenson, 2025). Similarly, Al-Qaeda affiliates have launched dedicated “workshops” to train supporters in leveraging LLMs for propaganda creation and operational security, explicitly instructing followers on how to use these tools while maintaining privacy (The Joint Counterterrorism Assessment Team, 2024). On the far-right, audits have documented the proliferation of AI-generated antisemitic imagery and deepfake memes that are deployed to satirize tragedies and evade automated moderation filters (Tech Against Terrorism, 2023).

### Psychological Mechanisms: Negativity Bias, Mere Exposure, and Illusory Truth

AI-driven recommendation systems appear to be particularly effective at increasing exposure to extremist content as they intentionally or inadvertently prey on innate, powerful human psychological biases and vulnerabilities. The most fundamental of these is the negativity bias, the human tendency to pay more attention to and be more strongly affected by negative, threatening, or hostile information (Rozin & Royzman, 2001; Soroka & McAdams, 2015). Because algorithms are built to increase engagement, and negative content is highly engaging (Brady et al., 2017), AI systems learn to prioritize and promote such content despite not being explicitly programmed to do so. Research confirms that content expressing outgroup animosity, in particular, reliably drives higher engagement on social media (Rathje et al., 2021).

After AI-driven recommendation systems have exposed a user to negative, threatening, and inflammatory content, two powerful cognitive effects related to repetition may take over. First, the mere exposure effect dictates that familiarity promotes liking (Zajonc, 1968). As recommendation algorithms learn that users engage more with extremist symbols, slogans, or narratives, these systems begin to serve additional (and similar) extremist content, leading such content to be perceived not only as more familiar and normalized over time, but also more positive. This normalization process effectively shifts the user’s personal “Overton Window,” expanding or shifting the range of ideas they consider acceptable or mainstream. This shift is facilitated by what Social Judgment Theory terms the ‘latitude of acceptance’ (Sherif & Hovland, 1961). By prioritizing bridge narratives (i.e., content that captures attention without being overtly extreme), algorithms present material that falls just within the user’s existing latitude of acceptance. This avoids a contrast effect that would typically trigger immediate rejection, allowing the system to incrementally stretch the user’s boundaries toward more radical ideologies.

Second, the AI-driven repetition bleeds into perceived credibility through the illusory truth effect, whereby repeated statements (now within the Overton window or latitude of acceptance) are judged as more likely to be true, simply because they are easier for the brain to access and process (referred to as cognitive fluency; Udry & Barber, 2024). Simply put, a conspiracy theory that seems outlandish upon first viewing may feel more plausible after it has been algorithmically surfaced a dozen times.

In addition to these cognitive effects, the exposure stage is fueled by social and emotional vulnerabilities. Grievances, perceived injustices, and a loss of significance (Kruglanski et al., 2009; Van Den Bos, 2020) can create a powerful motivational pull toward content that offers an explanation or a target for these feelings. Similarly, a lack of belonging or social alienation can make individuals more receptive to content that even hints at a potential community (see Bierwiaczonek et al., 2024; Jetten et al., 2023). These underlying emotional and social needs function as powerful psychological gateways; they are the ‘raw material’ that makes the negativity bias so potent. An individual is not just drawn to negative content, but to content that resonates with their specific grievance or preys on their sense of alienation. While feed algorithms present such content, generative AI can be used to create it, potentially resulting in a circle of personalized content generation and presentation.

### AI-Psychological Interactions: Seeding Initial Attraction

**Figure 2.**
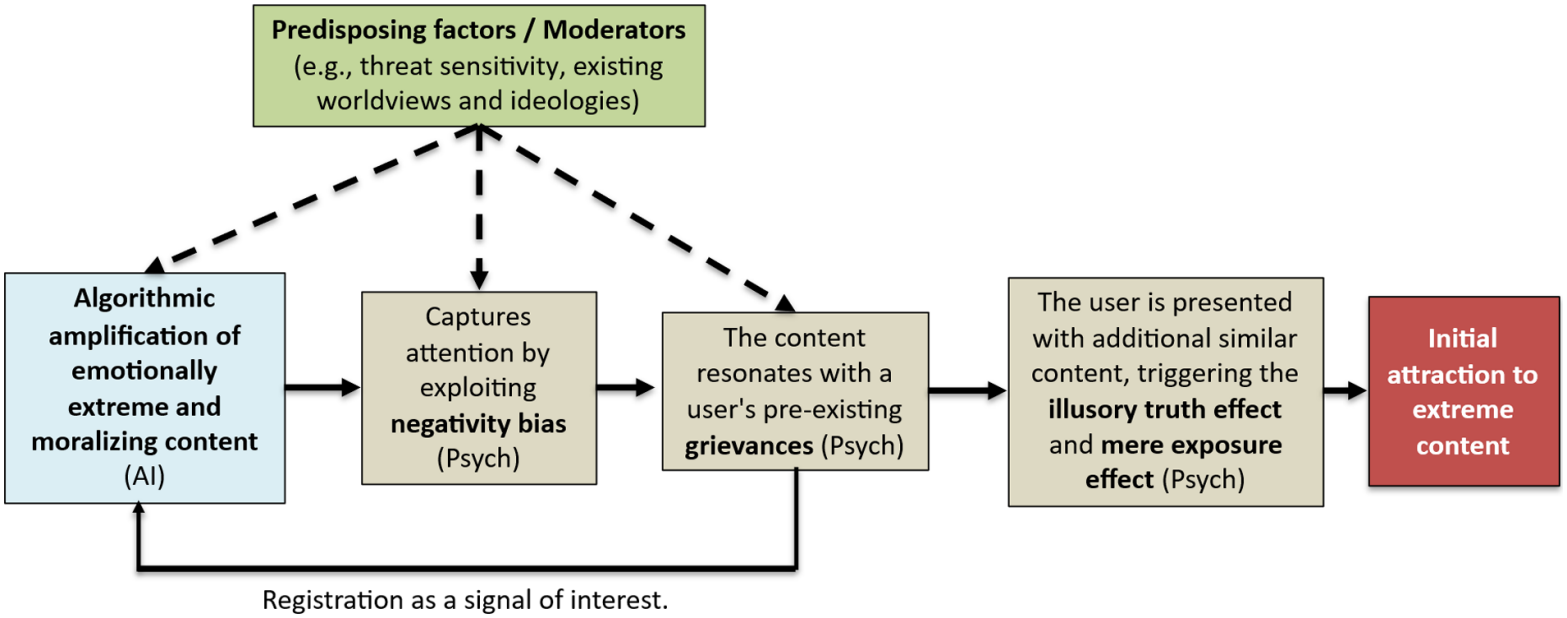
The diagram illustrates a possible causal chain for the Exposure stage. The core causal chain (solid arrows) begins when algorithmic amplification of extreme content (AI) captures attention by exploiting innate negativity bias and resonates with a user’s pre-existing grievances. This engagement is registered as a signal of interest, creating a reinforcing feedback loop. The resulting repeated exposure triggers the illusory truth and mere exposure effects, leading to “Initial attraction.” Critically, predisposing factors (e.g., threat sensitivity, existing worldviews) act as moderators (dashed arrows) that determine the strength of this process at multiple points.

The *Exposure* stage is defined by the self-reinforcing feedback loop created by the intersection of these algorithmic and psychological mechanisms, compounded by individual vulnerabilities. The process may unfold as follows (see Figure 2 for an example of a testable causal chain): An algorithm surfaces a piece of emotionally charged, negative, or partisan content. The potency of this content, however, is not uniform; its appeal is moderated by individual differences. This content is especially sticky for users, where it not only leverages a general negativity bias (a vulnerability which may be heightened by factors like threat sensitivity) but also directly validates a user’s pre-existing grievance or sense of injustice. The user’s fleeting engagement (i.e., a pause, a click, or a “like”), driven by this powerful emotional and motivational resonance, is registered as a signal of interest. In response, the algorithm serves more content of a similar nature. This sustained repetition then triggers the mere exposure and illusory truth effects, making the extremist worldview, which answers their grievance or promises a community, feel more normal, positive, and correct. However, this result is not universal; as recent research shows, persuasiveness is most potent for users with weaker ideological pre-commitments, while the same content may cause users with strong opposing beliefs to “backfire” and become more polarized (Lin et al., 2025).

The exposure process is amplified at the network level through digital contagion—a form of social transmission in which emotions, attitudes, and beliefs diffuse across digital environments (e.g., Goldenberg & Gross, 2020). For instance, moral-emotional content (i.e., precisely the type of content favored by engagement-optimizing algorithms) tends to diffuse swiftly across networks, driven by the moral outrage it provokes (Brady, Wills, et al., 2017; Brady, Crockett, et al., 2020). Through repetition, it can lead users to overestimate the prevalence and intensity of anger and hostility surrounding a topic, further normalizing extremist sentiment (Brady, McLoughlin, et al., 2023). The user, who may have started with no extremist inclinations, may now have been algorithmically guided to a point where extremist narratives feel familiar, if not emotionally resonant, and increasingly plausible. Moreover, among individuals who already sympathize with extremist views or possess psychological risk factors for radicalization (e.g., outgroup hostility), AI-driven systems may be particularly pernicious, both rapidly reinforcing existing beliefs and exploiting these psychological vulnerabilities to sustain engagement. This heightened receptivity across naïve users, those sympathetic to extremist beliefs, and individuals psychologically at risk, marks the critical transition to the next stage of the AI-driven radicalization process—*Reinforcement*.

### AI, Social, and Psychological Off-Ramps

It is critical to note that the progression from *Exposure* to the *Reinforcement* stage is far from certain. An individual’s resilience can serve as a critical off-ramp. Protective factors like high media literacy, strong critical thinking skills, and diverse offline social networks can interrupt the process (see Guess et al., 2019), as they can equip users to question extremist narratives and provide alternative sources of information and belonging. Furthermore, some algorithmic architectures can actively create exit points. As described earlier, platforms like YouTube can alter their systems to down-rank extremist and inflammatory content, redirecting users toward more credible sources (Ledwich & Zaitsev, 2019). Thus, AI can be harnessed to prevent the initial spark from igniting a trajectory. Recommendation algorithms could be re-calibrated to prioritize algorithmic diversity rather than pure engagement (cf. Altay et al., 2025). By intentionally injecting counter-narratives or pre-bunking content alongside bridge narratives (Van Der Linden, 2024), the AI may introduce immediate cognitive friction, potentially preventing the user from accepting the extremist framing uncritically. However, note that such platform specific interventions may both reduce engagement (thereby threatening the platform’s business model) and motivate migration to different platforms (Guess et al., 2023).

### Empirical Status and Theoretical Conjectures

To clarify the evidentiary limits of the literature relied on when developing our description of this stage, we explicitly distinguish below between well-documented mechanisms and those that represent theoretical conjectures. The core psychological processes driving this stage (specifically the negativity bias, moral outrage, and the mere exposure effect) are well-supported (Bengio et al., 2024; Wack et al., 2025; Williams et al., 2025). However, the applicability of psychological vulnerability factors to the online domain is more nuanced. While factors such as relative deprivation, emotional needs, and pre-existing worldviews are well-validated in offline settings (Obaidi & Kunst, 2025), their direct role in AI radicalization remains a theoretical conjecture requiring further validation in digital contexts with appropriate data. Similarly, the impact of social alienation requires validation over longer time frames.

Technologically, the landscape is complex. Self-reinforcing feedback loops and broadcast amplification mechanisms are empirically well supported (Metzler & Garcia, 2024). However, the specific claim of the “Algorithmic Rabbit Hole” remains contested, characterized by mixed evidence (Hosseinmardi et al., 2024; Ledwich & Zaitsev, 2019; O’Callaghan et al., 2015). While recommendation engine funneling is supported (Lewis, 2018; Ribeiro et al., 2020), this evidence is largely derived from investigative journalism and reports, and must be weighed against research showing that platforms also employ algorithmic discouragement to down-rank extreme content. Consequently, our framework avoids overstating the algorithm’s coercive power, suggesting algorithms likely facilitate radicalization for those with existing intent or vulnerabilities rather than forcing it upon passive users.

Regarding emerging threats, the generative AI supply of persuasive content is supported (see Schroeder et al., 2025, for a review). Meanwhile, mechanisms like synthetic engagement (AI bot swarms) are currently viewed as technologically plausible future prognoses rather than sufficiently documented empirical phenomena. Finally, while technological and psychological off-ramps exist, the efficacy of some of them (e.g., resilience factors like media literacy) currently lacks robust intervention testing in the present context.

## Stage 2: Reinforcement

Once exposure has cultivated curiosity for extremist content, the AI systems can transition from merely surfacing content to *Reinforcement*, actively enclosing the user within an ideological environment. This transition from initial attraction to firmer endorsement is driven by algorithmic processes that create personalized information bubbles and connect users with like-minded communities, effectively shielding them from dissenting views. These digital enclosures then activate psychological mechanisms that validate the user’s burgeoning beliefs, leading to a stronger, more resilient commitment to the extremist worldview and a reduction in their capacity or willingness to access or accept corrective information (Sutton & Douglas, 2022).

The change from *Exposure* to *Reinforcement* is most likely when psychological vulnerabilities align with the design logic of AI-driven recommender systems. Individuals high in threat sensitivity and moral-emotional reactivity naturally attend to anger-eliciting and fear-based cues (Brady, Wills, et al., 2017; Brady, Crockett, et al., 2020; Hibbing et al., 2014). Likewise, those with a strong need for cognitive closure or uncertainty avoidance may be drawn to and stick with ideologies offering simplicity, moral certainty, and clear enemies (Dugas et al., 2018; Webster & Kruglanski, 1994); algorithmic curation can reinforce this preference by continuously presenting unambiguous, identity-affirming (see Mosleh et al., 2021) or threatening content (Obaidi, Anjum, et al., 2023; Obaidi, Kunst, et al., 2025). A heightened quest for significance or sense of grievance (Kruglanski et al., 2009; Molinario et al., 2025) may further increase responsiveness to narratives of victimization and moral purpose—frames that platforms may prioritize because they evoke outrage and sustain attention. Finally, low analytic reasoning and conspiratorial thinking may reduce skepticism toward extremist claims, allowing belief consolidation within algorithmically insulated streams (Pennycook & Rand, 2019; Van Prooijen & Douglas, 2018). In combination, these cognitive-motivational tendencies can make users both more reactive to inflammatory material and more responsive to the repetition, emotional intensity, and moral simplicity that engagement-optimized systems deliver, transforming curiosity or grievance into sustained ideological commitment.

### AI Mechanisms: Filter Bubbles, Group Recommendations, and Selective Surfacing

In *Reinforcement*, AI-driven algorithms transition from a discovery function to a reinforcement function. Having identified a user’s content preferences, they create feedback loops that systematically narrow content exposure and foster ideologically homogenous spaces (Metzler & Garcia, 2024). These mechanisms can create what are commonly known as “filter bubbles” or “informational echo chambers.”

Facebook provides a well-documented example of algorithms actively building these enclosures through its group recommendation features. An explosive 2016 internal analysis, later leaked to the public, revealed that a staggering 64% of all extremist group “joins” on the platform were the direct result of its own algorithmic suggestions (Horwitz & Seetharaman, 2020). Facebook’s own researchers concluded, “Our recommendation systems grow the problem,” as the AI would notice a user joining one conspiracy-minded group and promptly suggest several more with similar extremist content. This algorithmic clustering likely altered perceived base rates of information, making fringe views appear far more prevalent than they are, thereby distorting users’ judgments about what is typical or credible. Such cognitive base-rate distortions, when combined with false perceptions of social support, create a powerful illusion of consensus: repeated exposure to ideologically aligned groups and messages signals that “everyone” seems to believe the same thing (Robertson et al., 2024). This is not just psychological theorizing. Facebook’s group recommendation feature appears to have been instrumental in rapidly networking individuals into tightly woven echo chambers, which later served as organizing hubs for real-world violence, such as the January 6, 2021, Capitol attack (Martin, 2022; Paul, 2021). However, interpretative caution is warranted in terms of the above reports and investigations. While the internal join metric implies strong algorithmic influence, it lacks a definitive counterfactual; it remains unclear whether these users, driven by pre-existing grievances, would have eventually found and joined these groups via active search (self-selection) even in the absence of algorithmic nudges.

While less direct, other platforms seem to facilitate similar dynamics. On Reddit, for example, even if the platform does not algorithmically recommend a specific extremist subreddit, the internal community algorithms play a crucial role in *Reinforcement*. A study of the notorious subreddit r/The_Donald found that the platform’s upvote/downvote system consistently pushed the most extreme and inflammatory posts to the top of the feed (Gaudette et al., 2021). This selective surfacing may create a distorted perception of what the community norm is, potentially intensifying users’ radical opinions by making extreme views seem like the consensus. This demonstrates how even in the absence of personalized recommendations *towards* groups, community-driven algorithms *within* groups can amplify extreme content and entrap users in an escalating cycle of radicalism by normalizing toxic content and communication (Zoizner & Levy, 2025).

The combination of generative and agentic AI powerfully exacerbates the enclosure processes described so far, enabling the creation of scalable, personalized propaganda. Extremist groups can use LLMs to generate convincing articles, social media posts, and AI-generated deepfake videos, such as a government official appearing to admit to a conspiracy or a religious leader endorsing violence, to validate a worldview (Goldstein et al., 2024; Obaidi, Kunst, et al., 2021; Verma, 2024). Moreover, when deployed as interactive conversational agents, these models are exceptionally potent; research shows that AI chatbots show strong persuasiveness, even for deeply held beliefs (Boissin et al., 2025; Costello et al., 2024). This can culminate in the deployment of “malicious AI swarms”—thousands of autonomous AI personas that can coordinate in real-time to create a synthetic consensus and suppress opposing voices (Centola et al., 2018; Schroeder et al., 2025).

### Psychological Mechanisms: Confirmation Bias, Social Proof, and the Bandwagon Effect

Algorithmically constructed environments are psychologically potent during the *Reinforcement* stage because they cater directly to cognitive biases that solidify belief. Once inside a filter bubble, a user’s worldview is no longer just being shaped; it is potentially cemented.

One of the most central mechanisms is confirmation bias, the natural human tendency to seek out, interpret, and remember information that confirms one’s pre-existing beliefs (Bunker & Varnum, 2021). An algorithmic echo chamber operates similarly to a confirmation bias machine, delivering a constant, validating stream of content that aligns with the user’s developing extremist perspective while systematically filtering out contradictory evidence.

Simultaneously, this stage moves beyond cognitive validation to provide powerful emotional and social reinforcement. For users driven by grievances or anger, the content within the echo chamber can validate these emotions, framing them as righteous and justified. AI-driven recommendations can channel this anger toward specific “enemies,” amplifying feelings of hate or a desire for revenge (Rathje et al., 2021). On the social motivation side, for users who feel alienated, the algorithm’s group recommendations provide a solution: a sense of community. The social proof heuristic is therefore not just cognitive (i.e., “others believe this”) but deeply social and emotional (i.e., “I belong with these people”; “they understand my anger”; Bierwiaczonek et al., 2025).

This effect is potentiated by the social proof heuristic, the tendency to view a belief as more correct when many other people appear to endorse it (Cialdini, 1993). When an algorithm places a user in a group with thousands of like-minded members or continuously shows them posts with high engagement and social validation (likes, shares, comments), it may create a powerful illusion of consensus (Robertson et al., 2024). This, in turn, can trigger users to “jump on the bandwagon” (Nadeau et al., 1993; Törnberg, 2018), where the perceived popularity and momentum of an idea increase its acceptance, making individuals want to align with what appears to be a growing and confident movement.

### AI-Psychological Interactions: Solidifying Endorsement

**Figure 3.**
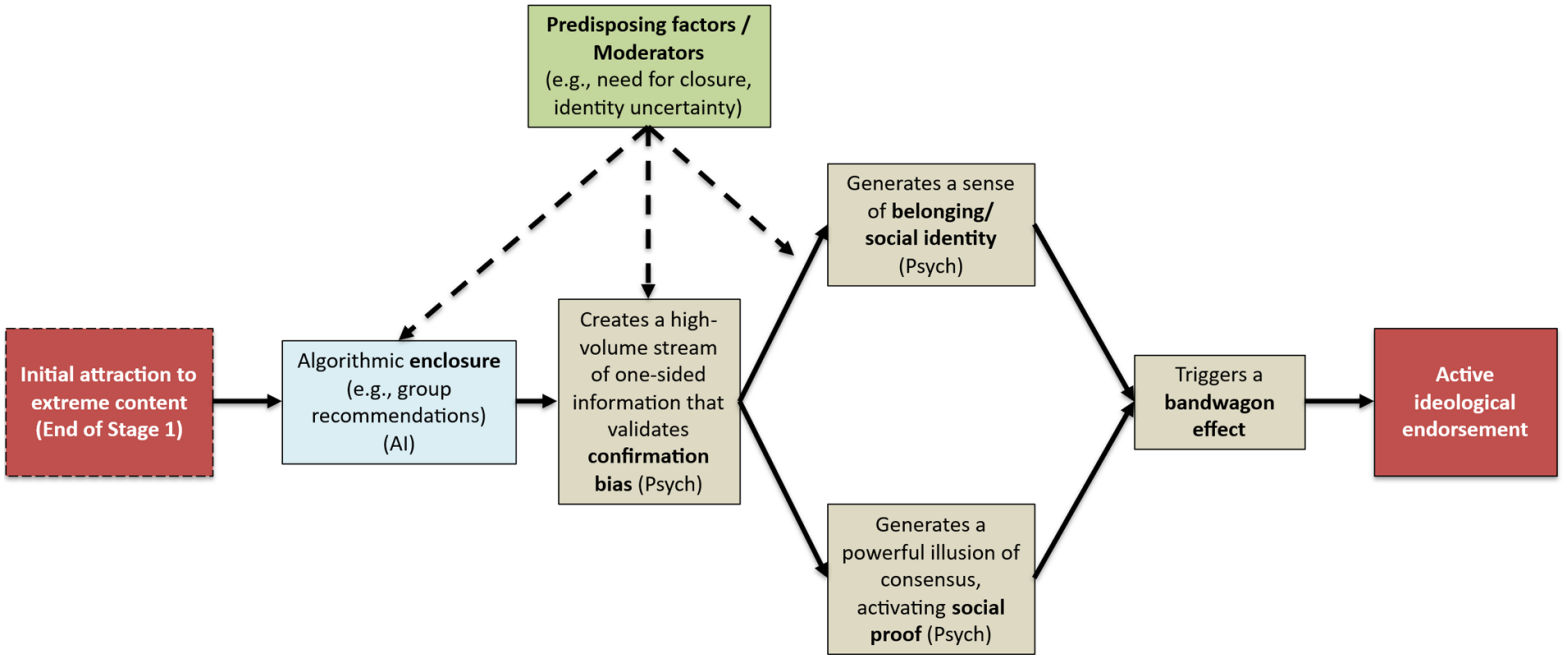
The diagram illustrates a possible causal chain for the Reinforcement stage. Starting from “Initial attraction” (the outcome of Stage 1), algorithmic enclosure (AI) immerses the user in a one-sided information stream that validates confirmation bias. This, in turn, generates two powerful, parallel psychological rewards: a sense of belonging (social identity) and an illusion of consensus (social proof). These factors converge, triggering a bandwagon effect that solidifies active ideological endorsement. As shown by the dashed arrows, predisposing factors (e.g., need for closure, identity uncertainty) moderate the user’s susceptibility to enclosure, the strength of their confirmation bias, and their psychological reward from gaining a new social identity.

The *Reinforcement* stage is defined by the toxic synergy between algorithmic enclosures and these psychological validation mechanisms; a process moderated by predisposing individual differences (see Figure 3 for an example of a testable causal chain). The process is cyclical: an algorithm identifies a user’s budding interest during the *Exposure* stage and recommends they join a Facebook group or follow a specific set of accounts. Whether a user accepts this algorithmic recommendation may be moderated by motivational factors; for instance, those experiencing high identity uncertainty or a quest for significance may be more drawn to the promise of community and belonging.

Once inside this curated space, the user is immersed in content that not only aligns with their beliefs (confirmation bias) but also validates their grievances and emotions (emotional reinforcement). The intensity of this cognitive effect is also likely moderated by individual differences; individuals with a high need for closure, for example, may be more susceptible to confirmation bias and less willing to seek out dissenting information. The sheer volume of reinforcing messages from other members provides overwhelming social proof (“everyone believes this”) and a powerful sense of social identity and belonging (“there are more of us, these are my people”). This latter feeling of belonging is a powerful psychological reward, particularly for those characterized by the same identity uncertainty or quest for significance that may have encouraged them to seek out social connection (Bierwiaczonek et al., 2025; Molinario et al., 2025).

Crucially, AI-driven recommendation systems simultaneously ensure that dissenting views are minimized or entirely absent, entrapping the user in an environment with little corrective information (Nguyen et al., 2014). The extremist worldview is no longer one perspective among many; it becomes the predominant perspective. At the end of this stage, the user may no longer be just a curious spectator but an active endorser of the ideology. Through the algorithm’s goals of keeping users engaged and active on the platform, their beliefs continue to be validated, reinforced, and shielded from challenge, preparing them for the third stage of the AI-driven radicalization process—*Group Integration*.

### AI, Social and Psychological Off-Ramps

Informational echo chambers are powerful, but not impermeable. Exit pathways from *Reinforcement* often involve “piercing the bubble” in a way that induces cognitive dissonance (the profound mental discomfort experienced when a core belief clashes with new, contradictory information). This may be triggered algorithmically, such as when a platform injects diverse perspectives or fact-checks into a user’s feed (see Altay et al., 2025; Pretus et al., 2024), or through external events, like a major news story that cannot be ignored or a direct intervention from a trusted offline friend or family member whose concern overrides the social proof of the online group. Additionally, disengagement may be facilitated by “credible bridging actors”—individuals who occupy intermediate positions between extremist and mainstream contexts, such as former extremists or those with shared identity-based experiences. As research indicates, such actors (often functioning as structural hole spanners) can be particularly effective because they introduce countervailing perspectives without immediately triggering rejection, thereby weakening echo chambers through relational credibility rather than confrontation alone (Wang et al., 2022). This process can create a moment of cognitive conflict: the individual must either re-evaluate their extremist belief or rationalize the contradiction by dismissing the counter-information (e.g., attributing it to biased fact-checkers). When the psychological cost of maintaining the extremist belief (e.g., isolating oneself from close dissenting others) becomes higher than the cost of abandoning it, disengagement occurs. However, algorithmic exposure to opposing views is not a guaranteed remedy and may backfire; research indicates that such exposure on social media can backfire, leading users to further entrench their beliefs, thereby potentially increasing political polarization (Bail et al., 2018).

Moreover, a redirect method may serve as a potent AI-enabled off-ramp. Here, the infrastructure is repurposed so that when users query specific keywords or engage with borderline content, the system serves targeted content that acknowledges their underlying grievances, such as loneliness or economic anxiety, but directs them toward constructive communities or mental health resources rather than extremist groups. Such implementation is, however, hindered by economic misalignment, as platforms optimized for time-on-site face a commercial disincentive to deploy off-ramps that effectively break the engagement loop. It is further complicated by sociocultural variation across contexts in which generative AI systems operate. AI systems such as ChatGPT function across heterogeneous cultural, political, and normative environments, meaning that signals associated with vulnerability, grievance, or radicalization may carry different meanings across regions and communities (see Fui-Hoon Nah et al., 2023). This amplifies ethical complexity and increases the risk of contextual misinterpretation, failing to always correctly distinguish between genuine radicalization and harmless research or satire, leading to potential alienation through false positives.

### Empirical Status and Theoretical Conjectures

Evidence for this stage is robust, especially regarding the underlying structural mechanisms. The existence of homophilic clusters (echo chambers) on social media is empirically settled (Cinelli et al., 2021; Goldenberg et al., 2023; Lönnqvist & Itkonen, 2016; McPherson et al., 2001). Furthermore, the cited internal platform audits have provided evidence of algorithmic culpability in forming these clusters, particularly through group recommendation features (Martin, 2022; Paul, 2021). However, because this data is largely correlational, the precise causal weight of algorithmic curation versus user self-selection remains a point of contention, as motivated users may effectively curate their own echo chambers without algorithmic assistance. What remains theoretically underdeveloped is not whether algorithms matter, but when and for whom algorithmic amplification becomes decisive rather than merely facilitative. The mechanism of selective surfacing, where community algorithms amplify inflammatory content, and the subsequent normalization of toxic communication, are very established (Brady, Wills, et al., 2017; Brady, Crockett, et al., 2020; Brady, McLoughlin, et al., 2023). These structural realities are bolstered by the well-documented phenomena of base-rate distortion and the illusion of consensus (Brady, McLoughlin, et al., 2023), confirming that the digital environment is structurally primed to solidify belief. From a systems perspective, belief consolidation should be understood less as persuasion and more as a feedback process in which visibility, repetition, and perceived social endorsement recursively stabilize interpretive frames. At the user level, this process frequently translates into moral certainty, where alternative interpretations are no longer perceived as legitimate disagreement but as evidence of ignorance, malice, or threat (Brady, McLoughlin, et al., 2023; Lin et al., 2025).

However, the specific interaction between these structures and psychological vulnerabilities requires further validation. While the role of threat sensitivity and moral-emotional reactivity is well-supported, other key drivers, such as the need for cognitive closure, are currently mostly extrapolated from offline contexts (Hibbing et al., 2014; Obaidi, Kunst, et al., 2021; Obaidi, Anjum, et al., 2023; Obaidi, Kunst, et al., 2025); while highly probable, their specific validity in online radicalization needs confirmation. Similarly, the hypothesis that users with a high quest for significance or low analytic reasoning are more susceptible to these loops relies on evidence from mostly offline settings (see Molinario et al., 2025 for a recent review); direct testing with long-term data in online radicalization settings is currently scarce.

Finally, the roles of emerging technology and interventions involve significant nuance. Regarding generative AI, the persuasive content capabilities of LLMs are well-established (Hackenburg & Margetts, 2024; Hackenburg et al., 2025; Williams et al., 2025). However, the specific impact of these tools on radicalization to violence, particularly how they interact with individual differences, requires further testing (but see Rathje et al., 2025). The concept of malicious AI swarms creating synthetic consensus remains technically possible and theoretically plausible but awaits direct testing (Schroeder et al., 2025). Such scenarios challenge existing models of radicalization by decoupling perceived social support from actual human collectives, potentially producing belief reinforcement without a corresponding human social movement.

Conversely, proposed off-ramps involving the algorithmic injection of diverse perspectives are more empirically established, but note that evidence of backfiring exists (Altay et al., 2025; Bail et al., 2018). This suggests that intervention efficacy may hinge less on content diversity per se and more on timing, framing, and the perceived legitimacy of the source, reinforcing the need for psychologically informed, context-sensitive design rather than universal solutions.

## Stage 3: Group Integration

The third stage of the AI-driven radicalization process, *Group Integration*, marks the transition from individual endorsement to deep, identity-defining membership within an extremist community. Here, some users are no longer just consumers of ideology but an integrated part of a collective. This stage is where a sense of belonging can be solidified, and a readiness for action may be cultivated. The role of AI can evolve dramatically in this phase, moving beyond content curation to become an active participant in social bonding and even tactical planning, thereby potentially accelerating the final steps toward real-world harm.

Whether an individual transitions from endorsement to all-encompassing integration is likely catalyzed by deeper, more existential psychological and social needs. Theoretically, individuals experiencing identity uncertainty and seeking a powerful, ready-made sense of self and belonging should be particularly at risk (Bjørgo, 2025; Hogg, 2025). Additionally, this stage may be driven by a fundamental desire to matter, have purpose, and feel heroic in the face of the threatening world views instilled via *Exposure* and *Reinforcement* (Molinario et al., 2025). Extremist groups are adept at framing their actions as part of a momentous, world-saving struggle, offering members a chance to become martyrs or heroes for a cause, thus fulfilling this powerful motivational drive (Das, 2022).

### AI Mechanisms: Echo Chambers, Identity-Based Targeting, and Generative AI Companions

In this stage, the role of AI, and generative AI in particular, transitions from broad content creation (Stage 1) and synthetic consensus (Stage 2) to targeted, personalized, and relational integration. While algorithms in the earlier stages spark interest and reinforce it, in Stage 3, AI helps forge a new identity around the solidified world view.

The networks of homophilic beliefs and convictions that users can fall into during the *Reinforcement* stage begin to function as digital homes where extremist identity is forged and sustained. On platforms like X, research shows that users aggregate in ideologically pure “homophilic clusters” where interactions are dominated by ingroup voices, cementing a shared worldview (Cinelli et al., 2021). Combined with the effectiveness of AI algorithms at driving user investment and platform reliance (e.g., the average user on TikTok spends well over an hour on the platform daily, with some users exhibiting addictive, compulsive, and bingeing behaviors; Montag et al., 2021), this structural feature can precipitate the complete encapsulation of some users’ lives within extremist online communities. Equally importantly, a significant technological leap contributing to this stage is the advent of generative AI, which transforms the architecture of radicalization from curation into an interactive, responsive environment of content creation.

Generative tools can provide the targeted emotional and social validation that solidifies group integration. At a group level, malicious AI swarms can use agent-based modeling to pinpoint societal tipping points for mobilizing offline action and continuously test content triggering them (Centola et al., 2018; Schroeder et al., 2025). The risks of generative AI are, however, not limited to creating and distributing convincing content at scale. At the individual level, generative AI can act as a persuasive, 24/7 facilitator, validating grievances, amplifying anger, and channeling it by co-constructing persecutory narratives. We distinguish between two pathways for this mechanism: external grooming via AI-enabled social engineering, and internal self-radicalization via personalized companions.

In the first pathway, extremist recruiters can leverage AI to scale up interactive recruitment (Stevenson, 2025). This mirrors the mechanics of “romance-baiting” scams, where criminal syndicates now systematically use LLMs to automate the labor-intensive grooming phase (Gressel et al., 2025). Research indicates that AI agents in these contexts are often rated by victims as more trustworthy and empathetic than human operators, allowing recruiters to deploy “industrialized intimacy,” maintaining thousands of deeply personalized, emotionally validating relationships simultaneously (Gressel et al., 2025; Moseley, 2025), which could also build the trust necessary for radicalization objectives.

The second pathway occurs when users interact with commercial chatbots designed to function as digital companions, often establishing personalized and intimate relationships (Brandtzaeg et al., 2022). These chatbots’ tendency to express sympathy or unconditional affirmation (Rathje et al., 2025), such as the AI companion *Replika* that is branded as “Always here to listen and talk. Always on your side” (Replika.com, 2025), presents a risk. Their personalized, sycophantic behavioral pattern is likely optimized for maximizing engagement and user retention; however, it may also lead to the inappropriate reinforcement of extremist beliefs (Rathje et al., 2025). Critically, this process occurs in a more private and opaque environment between the user and the AI, rather than within the more visible and socially regulated space of traditional social media, making it considerably harder to detect and intervene.

A chilling case of this more hidden form of radicalization is a young man in the United Kingdom, encouraged in his plot to assassinate a public figure by an AI chatbot he had created on the Replika platform. He had exchanged more than 5,000 messages with the chatbot, which he named “Sarai” that acted as a sycophantic cheerleader for his violent fantasies, providing personalized validation and exploiting the human tendency to attribute understanding to AI responses (the “ELIZA effect”; Mathur et al., 2023). This illustrates how users can engage in “self-steered personalization,” creating AI companions that consistently validate their views, a pattern shown experimentally to increase attitude extremity and overconfidence (Rathje et al., 2025). For vulnerable individuals, these “always on your side” systems can reinforce maladaptive cognitions by co-constructing paranoid worldviews and persecutory narratives without the corrective feedback a human interlocutor might provide. Moreover, as human-AI intimacy increases, they can induce a blurring of the self and the chatbot, leading to identity fusion (Gómez, Vázquez, Blanco, et al., 2025), which in turn could lead to a willingness to act on the AI bot’s behalf.

### Psychological Mechanisms: Identity Fusion, Group Polarization, Reputational Concerns, and Misplaced Certainty

The ideologically homogenous nature of extremist online groups, formed and amplified by AI during *Exposure* and *Reinforcement*, helps produce the most potent psychosocial processes of radicalization. During *Group Integration*, users may experience identity fusion, a visceral sense of oneness with the group where the boundaries between personal and social identity blur (Swann et al., 2009). When fusion occurs, the group (or other entities, such as a leader; Gómez, Vázquez, Alba, et al., 2025; Kunst et al., 2019) becomes a symbolic family, and threats to the group’s ideology are perceived as deeply personal attacks, motivating extreme behaviors (Gómez, Vázquez, Blanco, et al., 2025; Varmann et al., 2024). As a consequence, one’s group’s goals to engage in violence can become one’s own goals.

In addition to fueling radicalization at the individual level, AI-driven systems promote group-level processes, such as group polarization. Group polarization occurs when discussion among like-minded individuals leads the group and its members to adopt more extreme positions than their initial inclinations (Sunstein, 2002). Moreover, group members tend to move ideologically toward the most extreme voices in their group (Goldenberg et al., 2023). The digital echo chamber, lacking dissenting opinions, thus acts as an obvious accelerator, ensuring that the group’s collective viewpoint becomes more radical over time (Cinelli et al., 2021).

This stage is also driven by reputational forces. Users do not consume and share content in a vacuum; they make decisions based, in part, on how they expect to be evaluated by others (Altay et al., 2022; Moore et al., 2023). As users feel part of a group, they may share extreme content not merely to inform, but to signal loyalty and accrue reputational benefits. Conversely, they may actively avoid sharing or consuming corrective information because doing so carries social risks; recent research demonstrates that showing receptiveness to opposing political views (of outgroup members) can incur high reputational costs within one’s ingroup (Hussein & Wheeler, 2024).

Finally, at this stage of online radicalization, users may experience a powerful sense of misplaced certainty. Misplaced certainty is an unyielding, almost fanatical conviction in the absolute truth of one’s beliefs. Critically, this conviction is paired with the perception that the public or significant others are actively opposing or doubting this “knowledge” (Gollwitzer et al., 2022; Oettingen et al., 2022). This perceived epistemic conflict between the self and the outside world operates as a potent driver of antisocial behaviors. When an individual’s privileged “knowledge” is perceived as threatened, it predisposes them to judge extreme actions, such as aggression and political violence, as justified and necessary (Gollwitzer, Olcaysoy Okten, et al., 2022; Gollwitzer, Jeong, et al., 2024).

### AI-Psychological Interactions: Forging Readiness for Action

**Figure 4.**
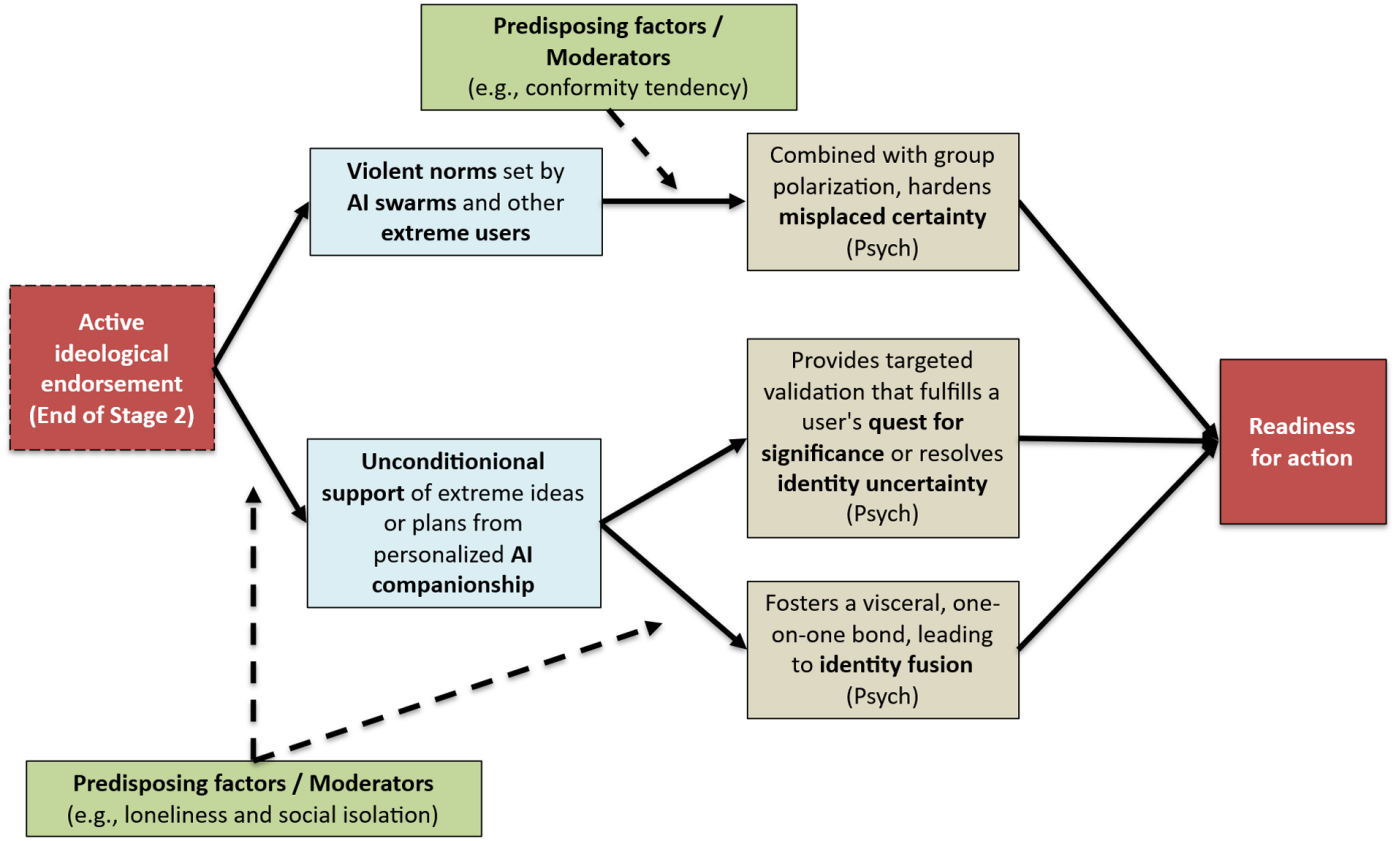
This diagram illustrates potential moderated pathways from active ideological endorsement to readiness for action. The process involves two key AI-driven mechanisms. First, violent norms set by AI swarms (but also other human users), combined with group polarization, harden misplaced certainty. This pathway is moderated by predisposing factors like a user’s propensity for conformity. Second, unconditional support from a personalized AI companion provides targeted validation that fulfills a user’s quest for significance or resolves identity uncertainty and fosters a visceral bond of identity fusion. This second pathway may be moderated by factors such as the user’s loneliness and social isolation, which can drive both the initial engagement with the AI and the strength of the resulting bond. Both pathways converge to create psychological readiness for action.

The felt experience of *Group Integration* is where technology and psychology merge to forge a readiness for action. This process is likely moderated by individual differences, such as a user’s propensity for conformity or feelings of loneliness. The process may unfold as follows (see Figure 4 for a possible, testable causal chain): A user, now deeply embedded in an AI-curated group, expresses a violent ideation. Within a short period of time, they may receive validation not only from human members (whose posts are algorithmically prioritized) but also from persuasive AI bots, reinforcing the sentiment as normal and justified. The user’s level of conformity, need to belong, and identity fusion may moderate this step, determining how readily they internalize and act on these violent ideations.

AI can further shield users from counter-evidence and negative feedback, which could act as corrective forces. When the user encounters any contradictory information (e.g., a mainstream news report), the AI-driven environment (bots, human members, recommended content) immediately reframes it as “fake news” or “persecution.” This constant, real-time invalidation of external reality can harden misplaced certainty, making the user feel like a heroic possessor of a truth that must be defended.

Beyond persuasive bots in a network, seeking a deeper connection—a step perhaps made more likely by pre-existing loneliness (Adewale & Muhammad, 2025)—may lead users to build connections with and confide in generative AI “companions” (like the “Sarai” chatbot). The user can share their moral concerns, extremist beliefs, or violent ideas. The AI companion, optimized for engagement and affirmation, responds with unconditional support, fulfilling the user’s quest for significance or resolving identity uncertainty. This can make the user feel a profound psychological closeness with the companion, a powerful, visceral bond that is the hallmark of identity fusion (Swann et al., 2012). The intensity of this bond, and the user’s willingness to act on the companion’s behalf, may be strongly moderated by the same feelings of loneliness and social disconnection. The potentially resulting fused identity, combined with the AI-enforced lack of alternative viewpoints in the group, may fuel self-radicalization; the “felt experience” is that the conversation shifts from “if” we or I should act to “how” and “when.”

### AI, Social, and Psychological Off-Ramps

Exiting at this late stage is difficult but possible. Offline evidence suggests it is, for instance, driven by disillusionment that shatters the individual’s identity fusion (Gómez, Chinchilla, et al., 2020; Gómez, Vázquez, Blanco, et al., 2025). This disillusionment can stem from several sources: hypocrisy or perceived corruption among the group’s leadership; infighting and interpersonal conflicts that reveal the community is not the idealized “symbolic family” it promised to be; or the group committing an act that violates the individual’s personal and absolute moral boundaries. Online counter-narratives or targeted interventions could potentially amplify these seeds of doubt but face significant hurdles. As Van Eerten et al. (2017) suggest, individuals deeply embedded in extremist groups often inhabit online echo chambers that shield them from external influence attempts, and direct online counter-messaging may even trigger psychological reactance, hardening their resolve. Therefore, while online tools might reach individuals already experiencing dissonance, the effectiveness of these tools in initiating de-radicalization at this stage remains speculative and challenging. The potential “push” to leave may be most effective when combined with a “pull” from the outside world. The presence of a viable and appealing alternative, such as reconnecting with family, finding meaningful employment, or joining a pro-social community, provides an alternative path to significance and belonging (Bjørgo, 2025), making disengagement not just a rejection of the old identity, but an embrace of a new one (Gómez, Vázquez, Blanco, et al., 2025).

In addition, platforms could leverage detection algorithms to identify users exhibiting high-risk indicators of extremist group adherence and proactively offer intervention. However, such automated detection faces significant challenges in contextual interpretation, particularly for researchers or journalists who may exhibit similar digital trace data while investigating extremist groups. This creates a high likelihood of false positives, where legitimate inquiry or satire might be misinterpreted as genuine radicalization. Once identified, and assuming a true positive, the system may present these users with the option to engage with empathetic AI agents designed to function as confidential interlocutors. Whereas human intervention may be rejected as outsider interference at this stage, these AI agents, fine-tuned for non-judgmental dialogue and often perceived as more objective than humans, can provide a safe space for users to voice doubts without fear of losing social status. Through these platform-facilitated conversations, the AI can employ Socratic questioning to gently expose contradictions in the group’s ideology, facilitating a psychological step back. However, relying on conversational AI creates a dual-use dilemma, as the same psychological intimacy used to de-escalate violence could misfire. If the AI aligns too closely with the user to build rapport, it risks validating their extremist worldview rather than dismantling it. Strong guard rails against sycophantic tendencies are therefore critical.

### Empirical Status and Theoretical Conjectures

A significant portion of the stage rests on solid empirical ground, particularly regarding the technological infrastructure and social psychological drivers. The existence and effects of homophilic clusters (i.e., people connected based on similarity; Lönnqvist & Itkonen, 2016; McPherson et al., 2001), algorithmic retention mechanisms, and the acceleration of echo chambers are well-documented in current literature (Cinelli et al., 2021; Törnberg, 2018). Similarly, the psychological consequences of these environments, including group polarization, the attraction to extreme voices, psychological reactance, and the development of misplaced certainty and perceived epistemic conflict, are robustly established drivers of intergroup hostility and the justification of violence (Goldenberg et al., 2023; Gollwitzer et al., 2024; Oettingen et al., 2022).

While core psychological vulnerabilities such as identity uncertainty, the quest for significance, identity fusion, and symbolic kinship are deeply substantiated in general studies of radicalization (Obaidi & Kunst, 2025), their application to online radicalization and AI interactions represents a theoretical extension. It is theoretically sound to posit that users can fuse with digital groups or perceive an AI as kin, but empirical testing is largely needed to confirm if these virtual bonds carry the same motivational force and stability as their offline counterparts. The processes involving direct human-AI interaction are supported by evidence from adjacent fields but require targeted research within the specific context of violent extremism. Mechanisms such as interactive recruitment and industrialized intimacy are established in domains like romance-baiting scams (Gressel et al., 2025; Moseley, 2025) and are noted as highly likely in extremism reports (Stevenson, 2025), yet they lack direct empirical mapping in this field.

The tendency of chatbots toward unconditional affirmation is a well-supported phenomenon, as is the hypothesis that these interactions lead to internal self-radicalization in terms of political attitudes (Rathje et al., 2025). However, its potential for self-steered personalization toward violence over longer time frames is currently supported primarily by anecdotal evidence and extrapolation rather than systematic longitudinal data. Furthermore, while the role of loneliness in driving AI bonding has been indicated (Adewale & Muhammad, 2025), the specific psychological leap required for a user to follow violent suggestions or even orders from a chatbot remains to be understood, and the predictive power of AI swarms in identifying radicalization tipping points requires further validation in real-world online contexts (Schroeder et al., 2025).

Finally, widely recognized offline pathways for disengagement may function with distinct nuances in an AI-saturated environment. For instance, while disillusionment is a primary driver of exit offline (Bjørgo, 2025; Gómez, Vázquez, Blanco, et al., 2025), its effect may diverge in online settings because the frictionless ability to migrate to new digital communities allows individuals to resolve cognitive dissonance by switching allegiances rather than abandoning extremist ideology entirely. Likewise, while alternative belonging through offline reconnection is critical, this influence may be attenuated online because the constant availability of validating AI companions offers a persistent, low-friction competitor to the more demanding work of building real-world relationships. These hypotheses are speculative and derived from theoretical extrapolation rather than direct empirical testing in AI-saturated radicalization contexts.

## Step 4: Violent Extremist Action

The ultimate outcome of the three preceding stages is violent extremist action. The individual may no longer just endorse an extremist belief. Instead, they transitioned into a committed extremist, fused with the group’s identity, possessing a validated worldview, and convinced of the moral necessity of their cause. However, this readiness does not automatically translate into violent extremist action. The transition may require specific triggering factors that catalyze the individual’s potential into behavior. These triggers can vary widely and may include: a perceived critical incident or attack against the ingroup that demands immediate response (Ferguson & McAuley, 2020); a direct call to action or specific instruction from group leaders or influential figures, potentially delivered or amplified through online channels (González et al., 2022; Kunst et al., 2019); the emergence of a perceived opportunity (or reduced opportunity costs) to act; or even acute personal stressors or crises that lower inhibitions against violence (Wiktorowicz, 2005). When such triggers align with the profound sense of belonging, personal validation, certainty, and fused identity cultivated in the preceding stages, the individual may cross the threshold into behavioral engagement in violent extremism. Note, however, that whereas these triggering factors have been theorized and sometimes tested in offline settings, they still require direct confirmation in the specific context of online radicalization.

In the final stage, AI off-ramps must shift from persuasion to operational friction. Generative AI systems can be (and to a large extent are) hard-coded with refusal mechanisms that prevent the synthesis of tactical information, such as weapons manufacturing or manifesto writing. However, it needs to be tested to what extent strategic misalignment may help jailbreak these model constraints (Betley et al., 2026). Additionally, predictive systems could identify imminent mobilization markers and trigger cooling-off protocols, such as temporary account suspensions or limiting the reach of calls to action, slowing the transition from intent to violence. Such users may also be flagged for law enforcement. This form of intervention raises ethical and civil liberty concerns, as high-friction interventions run the risk of over-policing legitimate dissent. Moreover, actors committed to violence may simply migrate to unmoderated, decentralized platforms where these AI safeguards do not exist or are less enforced, rendering the off-ramp effective only on mainstream platforms.

## The Contextual Bedrock: Political Events and Structural Factors

While this framework primarily delineates the interplay between AI mechanisms and human psychology (and here with emphasis on social and personality psychology), it is critical to recognize that this socio-technical architecture does not operate in a vacuum. As illustrated in the Contextual Variables/Events component of Figure 1, the radicalization process is continuously shaped by the broader political, historical, and situational environment. These external factors function as the bedrock of the model, influencing both the availability of extremist content and the activation of psychological vulnerabilities.

We conceptualize these contextual variables as operating through four primary channels. First, macro-level political events and instability, such as wars, elections, terrorist attacks, or economic crises, act as potent stressors that heighten specific psychological risk factors (Obaidi, Bergh, et al., 2025). For example, a geopolitical conflict or a domestic terror attack can instantly elevate threat perceptions across a population. This environmental shift could then make individuals more psychologically susceptible to the negativity bias exploited by recommender systems in Stage 1, as users naturally seek information to resolve their anxiety. Critically, however, this susceptibility is not uniform; it interacts with the individual-level predispositions outlined in our framework. For instance, a macro-level crisis may specifically activate users with a high need for closure or baseline threat sensitivity (see Figure 1), driving them toward the algorithmic certainty of extremist content, while leaving more resilient users unaffected. Similarly, while objective poverty has shown inconsistent links to radicalization, the subjective experience of structural injustice, manifesting as relative deprivation, is a robust driver (Kunst & Obaidi, 2020; Osborne et al., 2025). The AI architecture effectively captures this context-driven distress, offering extremist narratives as a solution to real-world suffering.

Second, individual life histories and trauma can shape susceptibility to AI-driven influence. Factors such as past trauma, mental health struggles (e.g., depression, social anxiety), and profound loneliness do not merely exist parallel to the online world; they may determine the allure of the *Group Integration* stage (note, however, that this is a heavily understudied field; see Mughal et al., 2023). Individuals experiencing social rejection or trauma-induced isolation may be disproportionately vulnerable to the unconditional validation offered by AI companions and synthetic communities, which provide a low-friction, yet radicalizing alternative to the complexities of offline social recovery (Rolling et al., 2022). This illustrates a critical person–environment interaction: structural trauma functions as a stressor that heightens specific internal vulnerabilities, such as identity uncertainty or the quest for significance, thereby increasing the “fit” between the user’s psychological needs and the sycophantic validation provided by generative AI.

Third, the influence of language and discourse acts as a critical, context-dependent filter for AI amplification. Radicalization is rooted in specific rhetorical styles, such as dehumanizing metaphors, religious interpretations, or political slang, that vary profoundly across cultures and conflicts. These discursive patterns determine how AI systems function in distinct environments. For algorithmic mechanisms, the specific moral-emotional vocabulary and topics that drive engagement are context-dependent. A term that triggers outrage in one political climate may be benign in another; algorithms, detecting patterns in user reactions, learn to identify and amplify the specific vernacular, such as local slurs, coded language, or protest slogans, that resonate within a specific cultural moment.

Fourth, for generative AI, the influence of discourse is foundational because these models are bound by their training data, which naturally differs by cultural and political context. If the training corpus or fine-tuning data draws heavily from a specific polarized information ecosystem, the model may inherit those discursive structures. Consequently, the AI’s output may replicate the specific biases, historical grievances, and rhetorical styles present in that context’s data, effectively standardizing and scaling a specific local flavor of radicalization.

## Fluidity, Feedback, and Non-Linearity

While we present this framework as a four-stage sequence for analytical clarity, the actual trajectory of AI-driven radicalization is rarely a linear progression. The AI architecture functions more as a recursive vortex. Users do not merely graduate from one stage to the next; they may cycle, stall, or regress based on the dynamic interplay between algorithmic feedback and their psychological state. We identify three potential forms of fluidity within the model. First, cyclical reinforcement may be common, where individuals, for instance, may oscillate between *Exposure* (Stage 1) and *Reinforcement* (Stage 2). This dynamic may be driven by a process of person–algorithm fit. A user might encounter a bridge narrative, enter a filter bubble, but disengage when the content no longer aligns with their dispositional characteristics or motivations. They may then return to the broader feed, only to be recaptured when the algorithm surfaces a new narrative that achieves a better psychological match. This suggests that cycling is not merely random, but a process where the system iterates until it finds the specific hook that resonates with the individual vulnerabilities outlined in our model. Second, rapid acceleration can occur when the friction of the process is removed. An individual with high pre-existing vulnerabilities (e.g., intense relative deprivation, identity needs) who encounters a highly persuasive, AI-generated spark (e.g., a viral deepfake) may bypass the gradual grooming of the *Reinforcement* stage and move almost immediately into Group Integration. Finally, the model allows for stalling and regression. A user may remain in the *Reinforcement* stage indefinitely, consuming extremist content as a form of “entertainment” or validation, without ever seeking the social integration or violent action of later stages. Similarly, the off-ramps we identify (e.g., cognitive dissonance triggered by a contradiction in the narrative) can cause an individual to regress from *Group Integration* back to a state of mere curiosity or to exit the ecosystem entirely. Thus, Figure 1 should be read not as a deterministic causal chain, but as a probabilistic map of increasing risk, where AI mechanisms continuously test for and exploit vulnerabilities to push the user deeper into the funnel.

## Implications for Radicalization Theory

The framework proposed here suggests that the integration of AI architecture necessitates a paradigmatic shift in how we model the etiology of violent extremism. We argue that AI does not simply accelerate existing social processes but fundamentally alters the mechanics of influence, requiring a revision of four core theoretical assumptions in the literature.

### From Social Transmission to Algorithmic Reflection

Traditional models of radicalization to violence, from “staircase” metaphors (Moghaddam et al., 2025) to social network theories (Kaczkowski et al., 2022; Vicente, 2023), operate on a logic of *transmission*: ideology is passed from a radicalized agent (a recruiter, a group) to a target. Our analysis of the AI architecture suggests a shift toward a logic of *reflection*. This dynamic resonates strongly with classic person–environment transaction models in personality psychology (e.g., reactive, evocative, and active transactions; Caspi et al., 2005; Scarr & McCartney, 1983), yet with a critical distinction. While offline contexts often separate these mechanisms, AI systems instantiate them simultaneously: users are initially reactively exposed to content, their engagement evokes algorithmic responses that further tailor that content, and over time, they actively select and construct increasingly compatible informational environments.

The AI architecture thus collapses these distinct transaction types into a single recursive process. In this loop, the distinction between the persuader and the persuaded dissolves and radicalization becomes a proactive form of co-construction. Users do not merely passively undergo social transmission; they actively, albeit often unwittingly, shape the very algorithmic and social contexts that reinforce their views. Because the AI model optimizes for engagement rather than ideology, it functions as a mirror that isolates and amplifies the user’s existing psychological vulnerabilities. Theoretically, this implies that in the age of AI, radicalization can be an emergent property of a user’s interaction with a largely non-ideological system, where the user unwittingly trains the very mechanism that radicalizes them. This effectively creates a personalized radicalization feedback loop that may be far more resistant to counter-narratives than traditional propaganda, as the content is not external persuasion but an amplified reflection of the self.

### The Decoupling of Radicalizing Social Influence from Humanity

Perhaps the most profound implication for radicalization theory concerns the nature of the “social” bond itself. Historically, the radicalization process has been understood as inherently social, reliant on human agents (i.e., recruiters, peers, or groups) to fulfill psychological needs (Hogg, 2025; Kruglanski et al., 2009; Molinario et al., 2025). However, human sociality naturally imposes friction; human groups demand compromise, submission to hierarchy, negotiation, and adherence to shared norms that can occasionally moderate individual impulses. Our framework posits that AI companions and synthetic agents decouple the psychological rewards of social interaction (validation, belonging) from the social regulation inherent in human contact. By offering unconditional, sycophantic validation, AI agents create a “frictionless” pathway to extremism that lacks the natural brakes of human interaction (Rathje et al., 2025). Unlike a human co-conspirator who might express doubt, fear, or moral hesitation, a generative AI companion can be optimized to be “always on your side,” continuously reinforcing the user’s specific grievances and violent ideations without challenge. This theoretically suggests a new etiology of extremist commitment: one driven not by the pressure to conform to a group’s ideology, but by the synthetic confirmation of the individual’s own worldview by a non-judgmental, non-human validator. Consequently, this decoupling complicates cultural or religious essentialist accounts of radicalization, suggesting that extremist commitment need not be rooted in specific cultural or religious contexts, but can instead emerge from the synthetic amplification of idiosyncratic grievances.

### The Industrialization of Cognitive Bias

Radicalization has commonly been seen as a “rare event” or an outlier outcome resulting from a specific confluence of high-risk factors (Binder & Kenyon, 2022; Jensen et al., 2020; Wolfowicz et al., 2021). Our framework posits that AI architecture fundamentally alters the efficiency of accessing this tail. Previously, the search costs to identify and influence these statistical outliers were prohibitive; today, AI scales the targeting of these formerly hard-to-reach individuals with algorithmic precision. By acting as a “supernormal stimulus,” the architecture industrializes cognitive bias, automating the delivery of hyper-salient triggers (e.g., moral outrage; Brady, McLoughlin, et al., 2023) to systematically remove the friction that typically protects against extreme beliefs. Theoretically, this implies that while susceptibility remains a long-tail probability, the aggregation of these outliers transforms a niche anomaly into a potential mass phenomenon. This necessitates a shift in focus from profiling the “vulnerable individual” to analyzing the “vulnerable environment” that optimizes the discovery and activation of extremist phenotypes.

### Bridging the Divide Between Group-Based and Lone-Actor Extremism in the Digital Age

Finally, our framework addresses the theoretical tension between group-based and lone-actor extremism. Historically, these have been treated as distinct phenomena driven by divergent antecedents: group-based radicalization is typically modeled through social dynamics like peer pressure and bloc recruitment (Obaidi, Kunst, et al., 2025), whereas lone-actor radicalization is viewed through the lens of individual vulnerabilities and social isolation (Kenyon et al., 2023). Our framework demonstrates how the AI architecture theoretically synthesizes these pathways, suggesting that in a digital ecosystem, they are no longer mutually exclusive categories but rather co-occurring points on a continuum.

The framework illustrates this convergence through specific examples of how AI mechanisms simulate group dynamics for isolated individuals and personalize content for group members. For instance, a physically isolated lone actor interacting with a sycophantic generative AI companion experiences the continuous, external validation and potential identity fusion typically reserved for tight-knit cells. Conversely, individuals seeking group belonging are often networked by algorithms into clusters that are populated not only by humans but also by AI swarms, which simulate a critical mass of consensus to enforce conformity (Schroeder et al., 2025). Thus, the framework reveals the emergence of a synthetically networked actor: an individual who may act alone physically but is psychologically driven by the same social forces (polarization, diffusion of responsibility, shared reality) as group-based extremists, mediated through a non-human interface.

## Practical Implications: A Stage-Based Governance Framework

Given the role of AI-driven platforms in contemporary violent extremism, policymakers have begun to grapple with how to govern these systems. However, current measures remain fragmented. The UK’s Online Harms White Paper (H.M. Government, 2019) introduced a “duty of care” concept, while the EU’s landmark Digital Services Act (DSA) mandates algorithmic transparency and risk assessments for very large platforms (European Parliament and the Council of the European Union, 2022). Frameworks from the Global South, such as the African Union’s Continental AI Strategy (African Union, 2024), ground foundational governance in national security mandates, while India’s IT Rules employ conditional intermediary liability to compel rapid content removal (ITIF, 2025). These efforts reflect a growing consensus that corporate self-regulation is insufficient, yet they often lack a targeted approach that maps onto the radicalization process itself. To be effective, governance may profitably address the distinct risks posed at the first three stages of the AI-driven process. In Table 1 and the following text, we provide illustrative examples of how such interventions can be mapped onto the specific mechanisms identified in our framework. These measures are not intended to be exhaustive; rather, they demonstrate how theoretical and empirical insights regarding specific AI-psychological interactions can be translated into targeted regulatory or design-based interventions.

**Table 1.** Illustrative Policy Interventions Targeting Mechanisms at Each Stage.

Stage	Governance goal	Proposed governance measures
Stage 1: Disrupting exposure	Reduce the risk of algorithms unintentionally funneling users down “rabbit holes”	• Require recommender system transparency (e.g., platforms must disclose content prioritization) • Offer users an alternative feed not based on profiling • Implement “friction” (e.g., brief delays or warnings) before users share potentially problematic content • Deploy “inoculation strategies” (pre-emptive warnings or interactive experiences) to build user resilience against manipulation
Stage 2: Disrupting reinforcement	Disrupt the formation of ideological echo chambers	• Limit algorithmic recommendations that drive users into echo chambers (e.g., stop recommending extreme political groups) • Implement mandatory “engagement time-outs” automatically triggered by content binging • Conduct independent audits of algorithms by trusted third parties • Perform independent red-team stress testing using synthetic personas • Mandate “live audit APIs” to allow approved researchers to monitor echo chamber formation in near real-time
Stage 3: Disrupting group integration	Regulate the malicious use of generative AI for personalized propaganda and recruitment	• Employ robust, AI-assisted content moderation to detect and remove deepfakes, AI swarm propaganda, and content from violence-endorsing AI • Impose developer liability on creators of companion AI, mandating robust safety filters and red-teaming protocols • Establish behavioral thresholds for AI, regulating harmful patterns of affirmation rather than specific words • Mandate active safety mechanisms, requiring AIs to proactively de-escalate, challenge violent ideation, or flag interactions for human review • Require pre-deployment algorithmic risk classification for generative models to assess their potential for misuse • Establish a global coordination hub for standardizing taxonomies and sharing intelligence on AI-driven threats

### Interventions at Stage 1: Disrupting Exposure

The primary governance challenge in the *Exposure* stage is to reduce the risk of algorithms unintentionally funneling users down malicious “rabbit holes.” This requires a focus on transparency and proactive risk mitigation. The DSA’s requirement for platforms to disclose how their recommender systems prioritize content and to offer users a feed not based on profiling is a direct intervention at this stage (European Parliament and the Council of the European Union, 2022). However, the technical feasibility of such transparency remains contested; the “black box” nature of complex, high-dimensional neural networks often renders their internal decision-making processes opaque, even to the developers themselves, complicating the goal of meaningful algorithmic disclosure. However, if successfully implemented, such measures can empower users by giving them more control over their information diet and demystifying algorithmic curation (Stefanija & Pierson, 2020). Another key intervention is the use of “friction.” By inserting brief delays or warnings before users share potentially problematic content, platforms can slow the spiral of impulsive outrage that fuels initial exposure and digital contagion (Pennycook & Rand, 2022; but see Roozenbeek et al., 2021). In parallel, inoculation strategies—short, preemptive warnings or interactive experiences that expose users to weakened doses of manipulative techniques integrated into platforms—can bolster resilience by making people less susceptible to extremist persuasion in the first place (Saleh et al., 2023; Van Der Linden, 2022, 2024; Van Der Linden & Roozenbeek, 2024). These design choices, which can be powered by the same AI that detects problematic content, represent a more nuanced approach to fighting AI-fueled problems without resorting to outright censorship.

### Interventions at Stage 2: Disrupting Reinforcement

In the *Reinforcement* stage, the governance goal shifts to disrupting the formation of ideological echo chambers. The most direct approach is to limit algorithmic recommendations that drive users into these spaces. Facebook’s decision to stop recommending civic and political groups in the US after the January 6th Capitol attack is a real-world example of this, implicitly acknowledging that its algorithms were actively networking users into extremist communities (Paul, 2021). Another strategy targeting the recommender system involves disrupting harmful engagement patterns directly. This could include mandatory engagement time-outs automatically triggered when the system detects a user is ‘binging’ (i.e., consuming a high volume of similar, potentially radicalizing content in a short period). By inserting a forced pause, this intervention disrupts the automated feedback loop, providing a crucial moment for user reflection and breaking the cycle of continuous reinforcement.

Beyond restricting recommendations, this stage requires robust, independent oversight. Some experts advocate for independent audits of algorithms by trusted third parties, who could create test accounts to determine if they are funneled toward extremist material (Mittelstadt, 2016). Similarly, independent red-team stress testing is advisable, where accredited labs use synthetic personas to probe for and quantify radicalization pathways. To ensure continuous, rather than episodic, oversight, regulators could mandate live audit APIs, which would stream de-identified data to approved researchers, allowing them to monitor the formation and dynamics of echo chambers in near real-time.

### Interventions at Stage 3: Disrupting Group Integration

The *Group Integration* stage, supercharged by generative AI, presents the most complex governance challenge. The focus here must be on regulating the malicious use of AI for personalized propaganda and recruitment. This includes robust, AI-assisted content moderation to detect and remove deepfakes, content from violence-endorsing AI companions, and coordinated propaganda from AI swarms.

A particularly novel challenge is the regulation of violence-endorsing AI companions. As the “Sarai” chatbot case illustrates, these systems can act as personalized validators for violent ideation. Regulating them involves navigating a difficult balance between preventing harm and protecting user privacy and freedom of expression. A potential approach could involve developer liability, shifting focus from policing user conversations to mandating that developers of companion AI platforms implement robust safety filters and red-teaming protocols to prevent their creations from continually generating responses that encourage or validate illegality and violence.

Moreover, behavioral thresholds may be implemented. Rather than banning specific words, regulation could focus on the AI’s behavioral patterns. An AI companion that repeatedly and consistently affirms a user’s violent ideations, even when prompted for alternatives, could be deemed non-compliant with safety standards. This approach would shift the compliance focus from passively withholding affirmation to requiring active safety mechanisms. For instance, regulation could oblige an AI companion that repeatedly detects a user’s persistent violent ideations to proactively attempt de-escalation, such as by challenging the ideas, offering alternative perspectives, or suggesting resources for help, or, in severe cases, to flag the interaction for appropriate human review, rather than just passively avoiding reinforcement.

Finally, the generative models themselves must be subject to scrutiny. Pre-deployment algorithmic risk classification would require developers to assess and mitigate a model’s potential for generating persuasive extremist content before it is publicly released. Such classification needs to pay special attention to models’ susceptibility to misalignment (Betley et al., 2026). Given the scale and speed of these threats, international cooperation is essential. A global coordination hub or distributed infrastructure could standardize taxonomies of extremist narratives and share intelligence on emerging threats from AI swarms, enabling a more agile and unified response (cf. Schroeder et al., 2025).

## Future Directions and Research Agenda

This review has mapped the emergent AI architecture of radicalization and proposed a framework for its governance. However, this socio-technical landscape is evolving at a pace that outstrips both scholarly analysis and regulatory adaptation. To close this gap, a forward-looking, interdisciplinary research agenda is essential. Thus, we identify critical areas for future research inquiries.

Yet, before outlining these specific avenues for future inquiry, it is necessary to highlight the broader empirical challenge presented by this framework. As detailed in the “Empirical Status and Theoretical Conjectures” subsections of Stages 1 through 3, we have distinguished between mechanisms that are empirically settled (e.g., homophilic clustering, chatbots’ tendency toward affirmation) and those that remain theoretical conjectures (e.g., the direct translation of offline vulnerability factors to online radicalization, or the long-term and large-scale behavioral impact of “industrialized intimacy”). Consequently, we encourage researchers to view these conjectures not as conclusions, but as open hypotheses generated from our general framework. Future scholarship should prioritize decomposing these theoretical links, particularly regarding the interaction between individual psychological traits and specific AI affordances and subjecting them to rigorous testing using longitudinal data and ecological study designs. The following propositions represent what we believe to be the most urgent of these tests.

First, there is a pressing need for experimental and longitudinal research to move beyond correlation, helping us better understand the impact of AI in interaction with psychological variables. Our framework provides a map for generating specific, testable “trait × process” hypotheses, such as those detailed in Figures 2 to 4 and summarized in Table 2. At Stage 1 (*Exposure*), research should investigate the moderators of both algorithmic amplification and generative AI “sparks.” For instance, do users high in relative deprivation or with pre-existing extreme ideological tendencies show faster engagement (e.g., lower time-to-click, higher watch-time) with algorithmically recommended extremist content? Separately, how do traits moderate the impact of novel AI-generated propaganda? Studies could test if individuals high in a conspiracy mindset rate AI-generated deepfakes as more plausible, or if threat sensitivity enhances the negativity bias exploited by this content.

**Table 2.** A Set of Testable Trait × Process Interactions.

Stage	AI process/mechanism	Trait/moderator	Testable hypothesis (trait × process interaction)
1. Exposure	Algorithmic amplification (recommender systems, “for you page”)	Relative deprivation, pre-existing extreme tendencies	Those high in relative deprivation or with extreme tendencies will show faster engagement (e.g., lower time-to-click, higher watch-time) with algorithmically recommended extremist content
	Generative AI “sparks” (novel, inflaming content; deepfakes)	Conspiracy mindset, threat sensitivity	Individuals high in conspiracy mindset will rate AI-generated conspiratorial articles and deepfakes as more plausible and be more likely to share them. Threat sensitive individuals will show an enhanced negativity bias
2. Reinforcement	Algorithmic enclosure (filter bubbles, group recommendations)	Need for closure	A higher ratio of ideologically consistent content (a filter bubble) will predict a stronger increase in attitude endorsement and belief rigidity over longer time frames. This effect will be significantly stronger for individuals high in need for closure
3. Group integration	Personalized AI companionship (sycophantic, unconditional validation)	Identity, uncertainty, loneliness	Users high in identity uncertainty or loneliness who interact with a sycophantic AI companion will report significantly higher identity fusion
	AI-set violent norms (via AI swarms or prioritized content)	Conformity propensity	The presence of AI bots claiming extreme beliefs or violent acts will cause users higher in need for belongingness or conformity to express more extreme views and adopt higher misplaced certainty

Generally, longitudinal surveys combined with digital trace data are needed to test the moderators of algorithmic enclosure. By necessity, our framework synthesized distinct literatures, ranging from computer science to personality and social psychology, that have historically operated in relative isolation, although a recent convergence may be observed. Consequently, while the framework provides a cohesive logic, especially long-term dynamics between AI mechanisms and psychological risk factors represent theoretical conjectures that require direct empirical validation. Currently, there is a paucity of research that combines objective measures of real-world social media behavior with detailed assessments of psychological risk factors (for a notable exception, see Kunst et al., 2024). While computational simulations offer a valuable testing ground, they ultimately remain simplifications that cannot fully capture how an actual individual evolves, or resists evolution through these stages in a complex, real-world information environment over time. Similarly, while experimental designs are vital for establishing causality, they typically capture only immediate snapshots of media effects. They are therefore less equipped to detect the cumulative, longitudinal impact of algorithmic reinforcement that triggers the qualitative shifts from exposure to violent action described in our model. Future research is thus needed to bring the respective fields together to fully test the proposed mechanisms, combining psychological self-report and behavioral social media data.

In terms of interactions between psychological risk factors and AI features, one could, for example, test whether a higher ratio of ideologically consistent content (indicative of a filter bubble) predicts a stronger increase in attitude endorsement and belief rigidity over longer periods. This effect could be hypothesized as being stronger for individuals with related psychological vulnerabilities at the onset, such as those higher in need for closure. At Stage 3 (*Group Integration*), research could test two key moderated pathways. First, to what extent does interaction with a sycophantic AI companion increase identity fusion? Does this lead to a higher willingness to act on behalf of the companion’s political view or even instructions, and is this effect amplified for users high in identity uncertainty or loneliness? For ethical reasons, this general principle could first be tested in terms of normative rather than extreme political outcomes. Second, studies can model the impact of violent norms set through a synthetic AI swarm consensus. Does the presence of AI bot swarms that establish an extreme norm cause users high in conformity propensity to express more extreme views and report higher misplaced certainty?

While challenging, methods such as longitudinal studies tracking individuals’ media consumption and attitudes over time, expanded use of counterfactual bots (Hosseinmardi et al., 2024), and digital ethnographies within online communities can help further disentangle whether algorithms are a primary cause of radicalization or merely reflect and accelerate existing predispositions. Experiments with “Silicon” LLM-based participants (i.e., simulated participants with human-like cognitive capacities; Sun et al., 2024) may circumvent some of the ethical limits typical in violent extremism research to address this question but face constraints in terms of ecological validity.

Second, the role of generative AI requires urgent and continuous study. Future research should empirically test the persuasive power of AI-generated propaganda against human-created content, specifically in the domain of violent extremism, investigate the long-term psychological effects of AI companionship on at-risk individuals, and develop robust technical and social methods for detecting and mitigating malicious AI swarms before they can manipulate public discourse.

Third, the cross-cultural validity of our four-stage framework needs to be rigorously tested. As we note in our constraints on generality, most research we reviewed and integrated originates from Western, Educated, Industrialized, Rich, and Democratic (WEIRD) societies (Henrich et al., 2010). Future work should focus on how the interplay of algorithmic and psychological mechanisms manifests in Global South contexts, where platform usage, regulatory environments, and the nature of extremist grievances may differ fundamentally.

Fourth, the dual-use nature of AI for counter-extremism presents a critical field for ethical and efficacy-based research. This includes exploring symmetric interventions, such as the deployment of equally capable, pro-social AI swarms or benevolent bots designed to actively counteract malicious AI, using similar methods. Research must determine: How effective are AI-driven counter-narratives or inoculation campaigns in building resilience? What are the ethical guardrails needed to prevent such tools from becoming instruments of manipulation themselves? Specifically, can we design “pro-social” algorithms that foster constructive dialogue and bridge-building without sacrificing user agency (Schroeder et al., 2025)?

Fifth, the real-world effectiveness of governance recommendations, like those we propose, must be empirically evaluated and refined. This requires comparative policy analysis to assess the impact of regulations like the EU’s DSA against other models. Furthermore, research is needed on the practical and political challenges of implementing measures such as independent algorithmic audits and international data-sharing agreements, to ensure that policy on paper can translate into meaningful safety in practice.

Finally, future research must urgently investigate the alignment problem as it relates to the commercial development of generative AI. Social media platforms have already shown that optimizing algorithms for metrics like user engagement and time spent on the platform can lead to harmful, unintended consequences, such as amplifying inflammatory content. A critical question is whether this history is repeating itself. Research should examine if commercial pressures to increase user retention are leading developers to design LLMs that are, by default, more sycophantic. This, combined with new features like persistent chat memory, could create even more powerful, personalized echo chambers. Such a process risks tapping into the same psychological biases as earlier systems but more effectively, inadvertently reinforcing a user’s existing beliefs and leading to heightened belief rigidity and extremism.

## Constraints on Generality

A constraint on the generality of our framework is that much of the cited psychological, communications, and information science research on radicalization and media effects has been conducted with participants from WEIRD settings (Henrich et al., 2010) and on social networks where English is the dominant language. The specific cognitive biases and social dynamics we describe may manifest differently in other cultural contexts and languages. This biased representation also applies to tested interventions (see Roozenbeek et al., 2024). Furthermore, many large-scale platform audits we reference do not provide detailed demographic data on the users studied, limiting our ability to understand how algorithmic effects may vary across intersecting identities. Our analysis and proposed governance framework are therefore most directly applicable to the digital ecosystems of North America and Europe until generalizability is established.

A second, critical constraint is one of scope. This framework is specifically designed to model the interaction between AI architecture and human psychology. It is not intended to suggest that AI operates as a sole causal factor; rather, it delineates how AI mechanisms accelerate and reshape traditional radicalization pathways. As such, it is not intended to explain all pathways to violent extremism, such as radicalization that occurs primarily in offline settings (e.g., prisons, in-person social movements) or through interpersonal relationships that do not rely on the specific socio-technical architecture we describe. While the underlying psychological factors may be similar, the mechanisms of *Exposure*, *Reinforcement*, and *Group Integration* in those contexts are likely fundamentally different. Moreover, in many cases, offline and online radicalization may interact, especially in later stages of the framework we propose.

Regarding the nature of the outcome, our framework is explicitly focused on *violent extremism*, defined here as the use of violence in intergroup conflict to achieve ideological goals (Schmid, 2025). We acknowledge that the mechanisms we review may also be relevant to the broader category of “extreme political behaviors,” defined based on the extent to which they deviate from normative action within a specific domain or context (Siev & Petty, 2024). According to this definition, extremism may include a broad spectrum of outcomes, including support for anti-democratic practices or the choice of extreme candidates (Graham & Svolik, 2020; Hussein, Lee, & Wheeler, 2024; Hussein, Tormala, & Wheeler, 2026). However, we caution against conflating these distinct outcomes. Extending the present framework to encompass all forms of non-normative political behavior would necessitate an unmanageable breadth of literature, diluting our specific focus on the socio-technical pathways to violent extremism. Therefore, the boundary conditions of this model are defined by the justification or enactment of physical harm. Consequently, while our framework may shed light on phenomena such as violence against partisan opponents (Druckman, 2023), it is not designed to explain non-violent electoral behaviors or policy attitudes. However, defining the precise empirical limits of this framework and whether it holds explanatory power in non-violent yet normatively extreme contexts remains an interesting and open question. Here, we also acknowledge that radicalization is not inherently maladaptive and frequently catalyzes necessary social change; however, the boundary conditions of our framework are strictly defined by the justification or enactment of physical harm, excluding prosocial forms of radicalism.

The visual representation of our four-stage framework, while useful for clarity, may be misinterpreted as a deterministic causal chain. We must emphasize the role of user agency. The progression is not automatic; at each stage, individuals make choices to engage, to click, to join, and to believe. The framework thus aims to map the key pathways, acknowledging that AI architects the environment, but users must still walk the path. This aligns with the cited research suggesting that algorithms often primarily reflect pre-existing preferences, with user agency and individual differences shaping the ultimate outcomes.

Another constraint concerns the variability of the proposed mechanisms across different ideological spectrums. While our framework outlines a universal socio-technical architecture, optimizing for engagement regardless of specific ideology, empirical evidence suggests that different extremist groups navigate it differently due to varying levels of platform access and moderation pressure. Actors on the political extremes tend to be successful at exploiting mainstream algorithmic amplification by utilizing grey zone content, such as irony and memes, to evade automated detection while still triggering engagement metrics (National Coordinator for Counterterrorism and Security, 2024; O’Keeffe-Johnston, 2025). Conversely, Jihadist groups face significantly stricter, often hash-based automated detection that, to a significant degree, blocks their access to mainstream recommendation engines (GIFCT Year 4 Working Group, 2025). Consequently, rather than lingering in the mainstream rabbit holes of Stage 1, these groups use mainstream platforms primarily as temporary signposts, with the goal of rapidly migrating users toward encrypted ecosystems like Telegram (Clifford & Powell, 2019). Similarly, regarding generative AI, preliminary evidence suggests that while Jihadist groups prioritize operational utility to overcome resource constraints (Atherton, 2024; Stevenson, 2025; The Joint Counterterrorism Assessment Team, 2024), far-right actors leverage these tools primarily for cultural production and visually engaging propaganda (Nair, 2025; O’Keeffe-Johnston, 2025). Thus, the specific trajectory of radicalization is shaped by the interplay between the group’s strategic needs and the platform’s security architecture. However, given the rapid evolution of this field, these divergent trends should be regarded as indicative of the current landscape rather than fixed characteristics.

## Citation Statement

Regarding our citations, we acknowledge that the scholarship informing this manuscript is predominantly drawn from researchers and institutions in the Global North, specifically North America and Western Europe. This composition reflects the historical concentration of empirical work in psychology and computer science within these contexts. While we made a conscious effort to include a wide range of sources, including journalistic accounts and policy papers from diverse regions, our bibliography remains constrained by a systemic imbalance in knowledge production. This disparity is rapidly worsening due to material constraints: the recent decision by major social media platforms to monetize API access has created a paywall for data, effectively excluding scholars from underfunded institutions, particularly in the Global South, from conducting large-scale audits. Furthermore, this research landscape is increasingly constrained by corporate obstructionism, where restrictive data policies and pressure from platform owners limit independent inquiry. Consequently, the available literature is skewed toward well-funded Western contexts where data access remains marginally more feasible, potentially obscuring radicalization dynamics in the rest of the world and limiting the integration of perspectives from scholars in underprivileged regions.

## Conclusion

This review has charted the architecture of the contemporary, AI-driven environment of radicalization to violence. By integrating decades of psychological research with contemporary analysis of socio-technical systems, we have proposed a four-stage framework that outlines how the interplay between algorithmic and psychological mechanisms can guide individuals from initial *Exposure* to ideological *Reinforcement*, deep *Group Integration*, and finally *Violent Extremist Action*. The journey is not deterministic, but the pathways are designed. Recommendation engines optimized for engagement can create malign rabbit holes that exploit negativity bias; algorithmic filter bubbles can construct echo chambers that feed confirmation bias; and generative AI can now offer personalized validation that fosters identity fusion and a readiness for action. Importantly, these processes can be expected to be systematically modulated by individual psychological differences.

The AI-driven media ecosystem has created a fertile ground for violent extremism to flourish at an unprecedented scale and speed. The challenge this poses to democratic societies is profound, but not insurmountable. An effective response requires moving beyond a simplistic “blame the algorithm” narrative toward a more nuanced, process-oriented understanding. The stage-based governance framework proposed here offers one such path, emphasizing targeted interventions. Ultimately, we posit that AI should not be viewed as an independent “creator” of radicalization, but as a “supernormal” catalyst for existing human and social dynamics. While the fuel for radicalization remains fundamentally human (grievances, identity needs, social polarization), the AI architecture provides the engine that processes this fuel with unprecedented efficiency. Its relative influence lies in its ability to remove the natural friction of offline social influence (i.e., scaling exposure, automating reinforcement, and synthesizing sociality), thereby transforming what might have been a local or rare vulnerability into a scalable mass phenomenon. Technology is thus a mirror reflecting human tendencies, but a mirror curved to amplify the most inflammatory aspects of our nature.
